# Mathematical Modeling of Proliferative Immune Response Initiated by Interactions Between Classical Antigen-Presenting Cells Under Joint Antagonistic IL-2 and IL-4 Signaling

**DOI:** 10.3389/fmolb.2022.777390

**Published:** 2022-01-28

**Authors:** Komlan Atitey, Benedict Anchang

**Affiliations:** Biostatistics and Computational Biology Branch, National Institute of Environmental Health Sciences, Research Triangle Park, NC, United States

**Keywords:** APC, immune response, dynamic proliferation of lymphocytes, joint IL-2 and IL-4 signaling, hybrid ODE models

## Abstract

During an adaptive immune response from pathogen invasion, multiple cytokines are produced by various immune cells interacting jointly at the cellular level to mediate several processes. For example, studies have shown that regulation of interleukin-4 (IL-4) correlates with interleukin-2 (IL-2) induced lymphocyte proliferation. This motivates the need to better understand and model the mechanisms driving the dynamic interplay of proliferation of lymphocytes with the complex interaction effects of cytokines during an immune response. To address this challenge, we adopt a hybrid computational approach comprising of continuous, discrete and stochastic non-linear model formulations to predict a system-level immune response as a function of multiple dependent signals and interacting agents including cytokines and targeted immune cells. We propose a hybrid ordinary differential equation-based (ODE) multicellular model system with a stochastic component of antigen microscopic states denoted as Multiscale Multicellular Quantitative Evaluator (MMQE) implemented using MATLAB. MMQE combines well-defined immune response network-based rules and ODE models to capture the complex dynamic interactions between the proliferation levels of different types of communicating lymphocyte agents mediated by joint regulation of IL-2 and IL-4 to predict the emergent global behavior of the system during an immune response. We model the activation of the immune system in terms of different activation protocols of helper T cells by the interplay of independent biological agents of classic antigen-presenting cells (APCs) and their joint activation which is confounded by the exposure time to external pathogens. MMQE quantifies the dynamics of lymphocyte proliferation during pathogen invasion as bivariate distributions of IL-2 and IL-4 concentration levels. Specifically, by varying activation agents such as dendritic cells (DC), B cells and their joint mechanism of activation, we quantify how lymphocyte activation and differentiation protocols boost the immune response against pathogen invasion mediated by a joint downregulation of IL-4 and upregulation of IL-2. We further compare our in-silico results to *in-vivo* and *in-vitro* experimental studies for validation. In general, MMQE combines intracellular and extracellular effects from multiple interacting systems into simpler dynamic behaviors for better interpretability. It can be used to aid engineering of anti-infection drugs or optimizing drug combination therapies against several diseases.

## Introduction

The immune system is composed of a large variety of cells and mediators that interact in a complex and dynamic network to protect the host against foreign pathogens and to simultaneously maintain tolerance towards self-antigens. This system is categorized into innate and adaptive immunity, both of which are key biological systems in generating acute and chronic inflammatory responses (Belardelli and Ferrantini, 2002). Nowadays, understanding the dynamics of the immune response during disease progression such as corona virus disease (COVID-19) including drug response represents a major challenge for biologists, physicians, and Engineers (Burnet, 1957; Thomas and Lawrence, 1959). Most mechanistic models applied to study immune response are population driven models. They include specific mechanisms/processes in a very simplified compartmental manner and do not account for joint extracellular and intracellular interactions at multiscale and multicellular levels (Handel et al., 2020). For example, the immune system contains many types of cells that communicate with each other mostly through proteins called cytokines. Cytokines act as messages that can promote, suppress, mediate and control immune and inflammatory responses at single-cell and multicellular levels. Moreover, complex interactions exist between cytokines, inflammation and the adaptive responses in maintaining homeostasis, health, and well-being (Elenkov et al., 2005). There is need to develop mathematical models that capture this level of complexity. We motivate the key players during an adaptive immune response and propose a hybrid ODE model with a stochastic component framework denoted as Multiscale Multicellular Quantitative Evaluator (MMQE) which combines network-based rules and ordinary differential equations-based (ODE) models to specifically study the dynamic proliferation structure of different types of communicating lymphocytes mediated by the joint signaling of key cytokines.

Proinflammatory and anti-inflammatory cytokines play a vital role in regulating the immune response under healthy and disease conditions. They bind to specific cell surface receptors to generate a cell signaling cascade that affects cell function (Minguet et al., 2007; Monaco et al., 2009; Arango Duque and Descoteaux, 2014). For example, cytokines regulate immune tolerance by targeting T cell activation and differentiation (Wu et al., 2014; Torres et al., 2017; Robertson et al., 2018) so that T cell immunity or tolerance to a particular antigen is regulated not only by T cell receptor (TCR) recognition, but also by various co-stimulatory and cytokine signals (Lacy and Stow, 2011). Despite the recent increase in single cell technologies accompanied by advanced mathematical models to study the intra or extracellular interactions of biological processes, multiscale dynamic modeling of the immune response between different cell types whenever there is intrusion of a foreign substance (pathogens) is still very challenging (Gog et al., 2015). Most experimental and mechanistic immune response models are optimized for single exposures. They focus on individual signaling pathways induced by independent cytokines and their specific receptors. It is still unclear how cells communicate between themselves in response to multiple simultaneous signals, which would be key for improving therapeutics for immune and inflammatory related diseases. Modelling the joint effects of interactions between cell growth factors such as cytokines IL-2 and IL-4 on the immune response with different activation protocols of the immune system would be key to optimizing the dynamic proliferation of the immune response against a potential pathogen. Thus, the proposed MMQE model takes into account not only the downregulation effect of IL-2 by IL-4 on the adaptive immune response but also different activation protocols of the immune system with classic APCs such as Dendritic cells, and B cells. Agent based models have been used to model viral clearance that emerges from interactions among immune cell agents (Folcik et al., 2011). In this study we characterize the resulting behavior (proliferation) from specific interactions of multiple cytokines and cells from pathogen invasion. This behavior is stochastic, as it does not follow linear rules (Chiacchio et al., 2014; Shinde and Kurhekar, 2020) making it appropriate to explore complex systems such as the immune system at multiple scales (e.g. lymphocytes, APCs or cytokine levels), which elucidates potential pathways leading to the targeted emergent behavior (Ozturk et al., 2018). Based on whether the emergent behavior is one that is to be avoided (suppression of the immune response), or one that is preferred, potential targeted cytokine interventions to the system can be studied to achieve the desired outcome against pathogen invasion.

The cytokine IL-2 is one of the key cytokines with pleiotropic effects on the immune system (Jiang et al., 2016). Initially, IL-2 was seen as the canonical T-cell growth factor (Kalia and Sarkar, 2018), inducing clonal expansion of T cells following antigen stimulation. It acts primarily as an autocrine growth factor but can also act in a paracrine fashion on nearby cells. In addition to its effects on CD4 and CD8 T cells, IL-2 also stimulates natural killer (NK) cells to proliferate and induce cytolytic activity when present at high levels and stimulates B cells to divide and produce antibody (Sharma and Das, 2018). The phenotypic expression of IL-2 in CD25-deficient mice has led to an appreciation of the importance of IL-2 in controlling immune response given that CD25 is the α -chain of IL-2’s receptor (IL-2R) which is expressed on activated T cells. It is thought that IL-2 acts primarily to enhance the development and maintenance of regulatory T cells (Tregs) but also suppresses T-helper 17 (Th17) effector cell differentiation as well as mediates cell death. Thus, IL-2 is critical to both the induction and the resolution of inflammatory immune response (Kalia and Sarkar, 2018).

Although the roles of cytokines such as Tumor Necrosis Factor alpha (TNF-α), IL-12, Interferon gamma (IFN-γ), and IL-10 in immunity and pathogenesis have been extensively studied, the role of interleukin-4 (IL-4) remains less understood (Wu et al., 2021). IL-4 is a multifunctional, immunoregulatory cytokine that functions as a master promoter of Th2 development, regulates a wide range of immune responses, and mediates many biological functions through its receptor IL-4R (Brown and Hural, 1997; Nelms et al., 1999). IL-4 targets many cell types to induce multiple effects, including cell proliferation, downregulation of Th1 responses, B cell differentiation, and immunoglobulins (Ig) class switching to produce IgE and IgG4/IgG1 (Acacia de Sa Pinheiro et al., 2007). IL-4 is also a profound neuromodulator and plays important roles in the physiology and immune functions of the nervous system, regulating inflammatory responses in the brain, and protecting the brain from Th1-induced encephalomyelitis, viral and toxoplasma encephalitis, and Alzheimer disease (Kirwin et al., 2006; Kiyota et al., 2010). In addition, IL-4 plays a role in antitumor immunity and autoimmunity and has been used as a therapeutic for autoimmune diseases and cancer (Kawakami et al., 2002; Ghoreschi et al., 2003).

Recently, an experimental study by Zhou et al. (2021) investigated the effects of IL-2 and IL-4 on a major type of suppressive regulatory T cells or Tregs. The study shows that when these cells are exposed to IL-2 and IL-4 simultaneously, the cytokine duo boost the production of new Tregs much more than using either cytokine alone (Zhou et al., 2021). Together, the cytokines also increased the production of another cytokine (IL-10), and over time, Treg cells producing IL-10 divide more frequently, leading to an even more robust ability to suppress overactive immune responses. IL-10 further inhibits anti-microbial immune responses, allowing fulminant and inevitably fatal infections to develop (Couper et al., 2008). Given that modeling efforts complement experimental platforms by providing an understanding of cells and cytokine dynamics and microenvironmental cues over time, attention needs to be given to computational modeling to provide a mechanistic insight into studying intra-cellular heterogeneity and interplay between cells and combinatorial functioning of cytokines such as IL-2 and IL-4 within their microenvironment. Since the immune response is contextual and specific (Nolte, 2018), it is important to study the interplay between the different levels of cytokines IL-2, and IL-4 production within the context of lymphocyte communication during an immune response.

**FIGURE 1:**
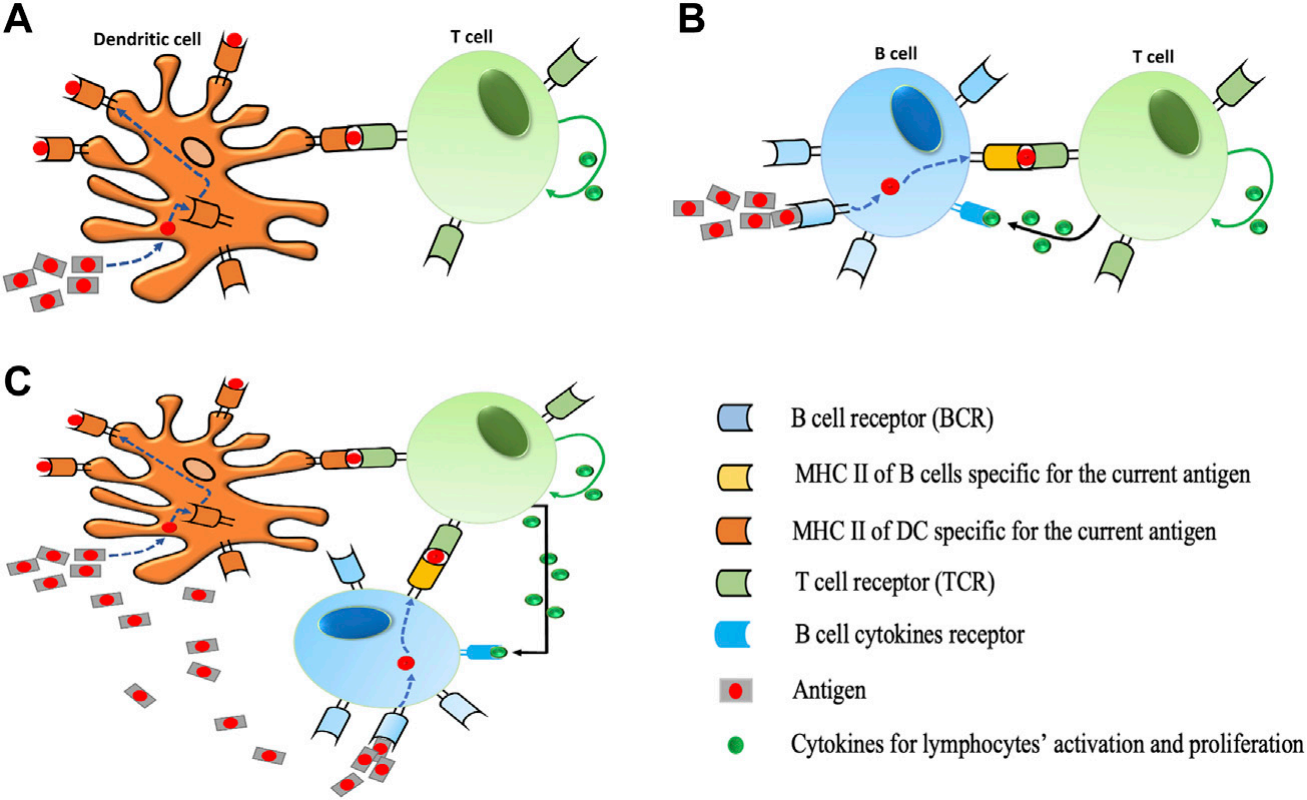
Different protocols for T cell activation. **(A)** During dendritic cell activation, dendritic cells (DCs) take up antigens, process them into short peptides, and present these on MHCs on their surfaces. The interaction between the T cell receptor (TCR) and the peptide-MHC complex (pMHC) at the interface between the T cell and the DC is the main event controlling the specificity of antigen recognition by T cells. T cells bind to pMHC *via* TCR. The proliferation of T cells is performed by specific cytokines (IL-2) in our model. **(B)** During B cell activation, a B cell is triggered when it encounters its matching antigen. The B cell engulfs the antigen and digests it and next displays the resulting antigen fragments bound to its unique MHC molecules. This interaction of antigens and MHC attracts the help of a mature matching T cell. Cytokines secreted by T cell activate B cells to differentiate into plasma B cells which results in the production of more antibodies. Likewise, the proliferation of B cells is performed by specific cytokines (IL-4) in our model). **(C)** Illustration of DC and B cells simultaneous activation. In the presence of a high number of antigens in the immune system, the activation of T cells would be triggered via both processes described in **(A)** and **(B)**.

T cells and B cells are the two major types of lymphocytes that are involved in triggering the adaptive immune response in the body. Before cytotoxic or helper T cells can kill or help their target cells, respectively, they must be activated to proliferate and differentiate into effector cells. This activation by binding on the surface of antigen-presenting cells (APCs) mediated by the Major Histocompatibility Complex (MHC) proteins (Roberts et al., 2002) normally occurs in peripheral lymphoid organs. The classical APCs are DCs and B cells which are effective in activating both the naïve and memory T cells in the presence of antigens (Gaudino and Kumar, 2019) (Figures 1A, B). Hence, specific recognition of antigens by B cell receptors induce B cells to proliferate and differentiate into plasma cells which provide antibodies against the antigens (Clark and Ledbetter, 1994). However, the production of antibodies by B cells requires assistance of activated T cells which depend on interactions between T cells and specialized APCs. Consequently, the immune system implements many different cell types and multiple intersecting molecular pathways and signals (Carbo et al., 2014) to achieve a desired response depending on the type of APC for activation.

Autoimmune diseases result from complex interactions among different immune cell types and cytokines. Recent studies about autoimmune diseases in animal models and in humans focus on lymphocyte activation and *in vitro* cytokine production. Since there are prospects for use of IL-2 and IL-4 in the treatment of autoimmune diseases or infections, the respective contribution of T cell cytokine production to the pathogenesis of autoimmune diseases is still a matter of debate (Carbo et al., 2014; Barrat and Su, 2019). Although agent based models are generally optimized for modeling spatial interactions (Figueredo et al., 2013), our current implementation of our MMQE model do not model space (distance) and how it would affect the simulation outcomes. With respect to the existing literature, the MMQE is designed to measure the performance of the immune response in terms of the proliferation of lymphocytes for different activation protocols dependent on the two classic APCs (DC and B cells) under the downregulation effect of IL-4 on IL-2. Additionally, MMQE considers the joint activation of DC and B cells given that both APCs can jointly activate the naïve or memory T cells in response to a high invasion of pathogens (Figure 1C). We further compare our analysis on recent studies on lymphocyte activation by APCs to validate our findings on how APCs boost better immune response in the treatment of autoimmune diseases to avoid inappropriate immune responses.

In the following sections, we first provide a detailed description of the MMQE for multiscale modeling. Specifically, we mathematically describe the modeling of pathogen variation, as well as the interactions between cytokine agents IL-2 and IL-4 supported by pertinent references. Moreover, we model the dynamics of activation, differentiation and proliferation of T cells and B cells. In the *Results* section, we present simulation and calibration results under a variety of activation protocols and varying concentrations of IL-2 and IL-4. Finally, we discuss implications of our work and provide some empirical validation. In a nutshell, MMQE employs a multiscale analysis to investigate different signaling regulatory mechanisms driven by multiple cytokines acting jointly to impact the proliferation of the immune response under different activation protocols.

## Materials and Methods

### Model Description

**FIGURE 2:**
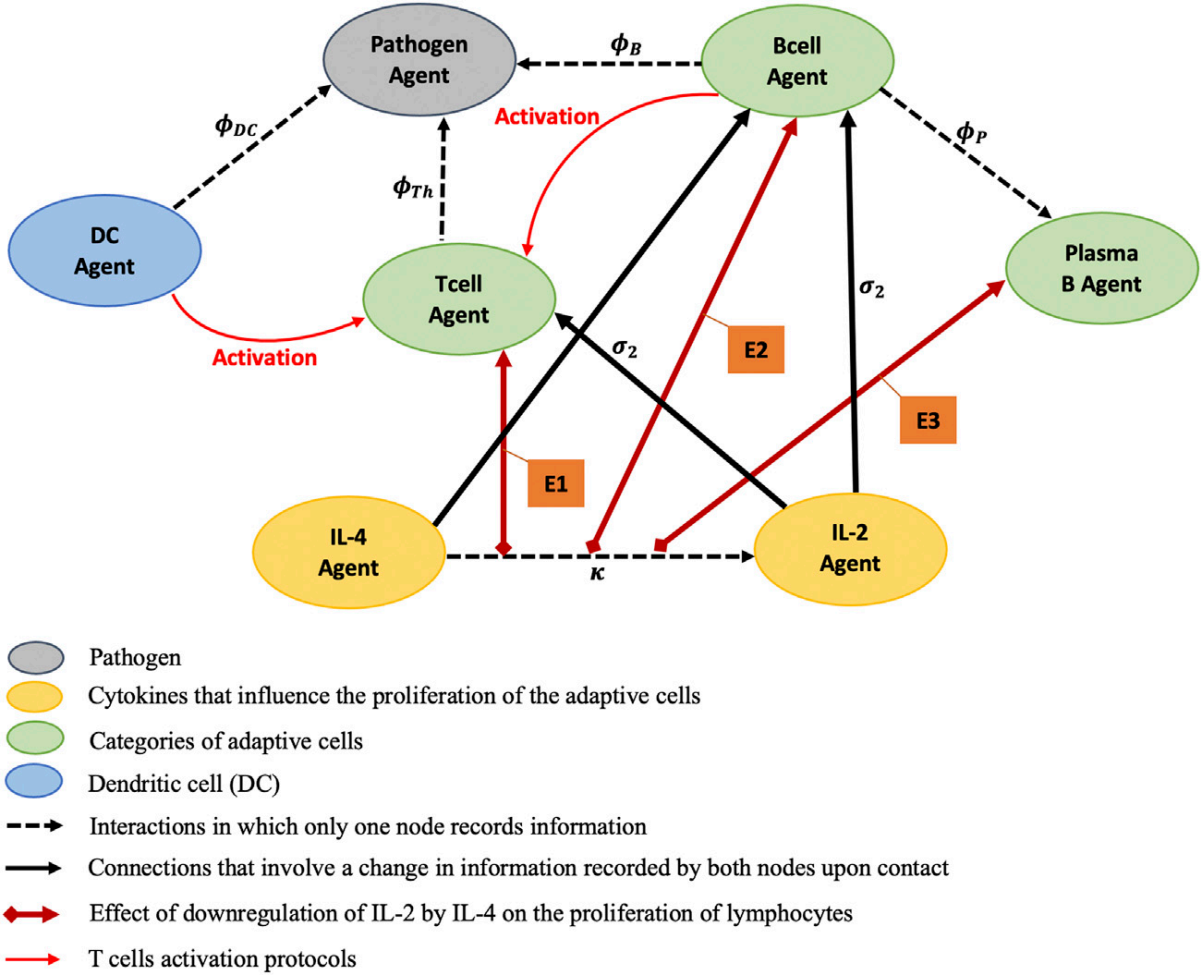
Static portrayal of agents and interactions of an adaptive immune response network. E1, E2, and E3 stand for modeling effects of the downregulation of IL-2 by IL-4 on the proliferations of T cell Agent, B cell Agent, and Plasma B cell Agent respectively. The red lines encode the T cell activation protocols controlled by DC and B cell Agents. The T cells proliferate at rates $\sigma_{2}$ , and become activated with rates $\phi_{\mathrm{Th}}$ as T helper cells. The B cells proliferate and become activated with rates $\sigma_{2}$ , and $\phi_{B}$ respectively. B cells differentiate into plasma B cells at a rate of $\phi_{P}$ . On activation, the dendritic cell matures into a highly effective antigen presenting cell (APC) and undergoes changes that enable it to activate T cells encountered in the lymph node with activation rate $\phi_{\mathrm{DC}}$ . The downregulation of IL-2 by IL-4 is processed with rate parameter κ .

The major key players and biological processes in the proposed MMQE (Figure 2) are: modeling the variations of the antigen (pathogen) agent, the joint interaction of cytokine agents IL-2 and IL-4, the dynamics of activation, proliferation and differentiation of B cell agent, the dynamics of activation and proliferation of T cell agent, the proliferation of plasma B cell agents and the activation of DC agent for overall immune system response. We also account for the life span of various lymphocytes during an immune response. For reasons of consistencies, we assign variables to agents involved in MMQE (Table 1).

**TABLE 1:** Lymphocytes, pathogens (antigens) and different APC agents in the MMQE.

Variable	Model
$A_{\mathrm{ag}}$	Microscopic state of antigens
$B_{t}$	B cell state at time *t*
$P_{t}$	Plasma B cell state at time *t*
$T_{t}$	T cell state at time *t*
$\Lambda_{t}$	State variable allocated to activation protocols controlled by classic APCs (DC and B cells)

### Modelling the Variation of Pathogens

One of the most effective and widespread strategies employed by pathogens in order to escape the human immune system is antigenic variation (Vink et al., 2012). This process can be described as a system that allows a pathogen to change its (surface) antigens that are presented to, and targeted by, the host’s adaptive immune system (Vink et al., 2012; Mosa, 2020). As a consequence of antigenic variation, subpopulations of antigenically distinct organisms arise within a population that are temporarily not recognized by the primary adaptive immune response (Barbour et al., 2006; Schmid-Siegert et al., 2017). This variability provides the pathogen with an extended window of opportunity to persist within a host or to infect a previously colonized host (Vink et al., 2012).

We define the dynamics of the pathogen agent distribution in the immune system as a random variable. Often, the distribution of activity per cell was found to be well described by a log normal distribution function (Koch, 1966) which is a type of probability distribution in which the logarithm of a variable is normally distributed (Crow and Shimizu, 1987; Balakrishnan and Chen, 1999). Correspondingly, theoretic modelling of cell survival as a function of mean activity per cell showed that survival curves differed substantially when the activity per cell was log normally distributed versus normally distributed (Limpert et al., 2001; Neti and Howell, 2006). Moreover, Sartwell’s model has been used to demonstrate that the incubation period for infectious diseases and genetic diseases fits a lognormal distribution (Horowitz et al., 2001). We therefore consider the variation of pathogens in this work as variations of the microscopic state of antigens $\left(A_{\mathrm{ag}}\right)$ , described by the log normalized density function (Mayer et al., 2015; Carignano and Dalchau, 2018) as:

$$\ln\left(A_{\mathrm{ag}}\right)∼N\left(\mu_{\mathrm{ag}},V_{\mathrm{ag}}\right)$$ (1)

where $\mu_{\mathrm{ag}}$ and $V_{\mathrm{ag}}$ stands for the mean and variance respectively of the variation.

### Cytokines IL-2 and IL-4 Interactions

We next illustrate with ODEs the complex interactions between the dynamics of cytokines IL-2 and IL-4. In general, cytokines are secreted proteins that carry intra and intercellular signals which regulate the immune response to pathogens or other pathological conditions (Chen et al., 2018; Muñoz-Carrillo et al., 2019). To effect their functions, cytokines must bind to specific extracellular receptors (Schreiber and Walter, 2010). Specifically, IL-2 and IL-4 are growth factors secreted by both T and B cells and demonstrate antagonistic marginal effects during immune response (Schreiber and Walter, 2010). The proliferation response to IL-2 stimulation is controlled by three factors: 1) the initial concentration of IL-2, 2) the density of IL-2R (receptor specific to IL-2) on the surface of the cells and 3) the duration of the cell exposure to IL-2 (Burke et al., 1997). On the other hand, studies on B cells have reported that IL-4 represents a growth and differentiation factor for the activated B cells and the IL-4 binding may have inhibitory effects on IL-2 induction (Castro et al., 1999). Therefore, a mathematical model based on the synthesis of all the known interactions is necessary to predict with high accuracy, the dynamics of T cell and B cell immune response. Let ***N*** and ***n*** be the bounded and unbounded number of cytokines such that $N_{2}$ and $N_{4}$ represent the bounded numbers of IL-2 and IL-4 to a specific cell with IL-2R and IL-4R receptors respectively. Likewise, $n_{2}$ and $n_{4}$ represent the unbounded numbers of IL-2 and IL-4 from IL-2R and IL-4R respectively. Based on the law of mass action, the quantitative terms for IL-2 and IL-4 binding can be derived and described by the following (Eqs 2–5).

$$\frac{dn_{4}}{\mathrm{dt}}=\phi_{4}-\theta_{4}I_{4}n_{4}+\varphi_{4}N_{4}-\gamma_{4}n_{4}$$ (2)

$$\frac{dN_{4}}{\mathrm{dt}}=\theta_{4}I_{4}n_{4}-\varphi_{4}N_{4}-\delta_{4}N_{4}$$ (3)

$$\frac{dn_{2}}{\mathrm{dt}}=\phi_{2}-\theta_{2}I_{2}n_{2}+\varphi_{2}N_{2}-\gamma_{2}n_{2}-\kappa N_{4}n_{2}$$ (4)

$$\frac{dN_{2}}{\mathrm{dt}}=\theta_{2}I_{2}n_{2}-\varphi_{2}N_{2}-\delta_{2}N_{2}$$ (5)

where, $I_{2}$ and $I_{4}$ represent the concentration of IL-2 and IL-4 respectively. The parameters $\phi_{4}$ and $\phi_{2}$ represent the production rates of unbound receptors IL-4R and IL-2R (Burke et al., 1997). $\varphi_{4}N_{4}$ performs the dissociation of IL-4 from IL-4R. The factor $\gamma_{4}n_{4}$ serves as the internalization of unbounded receptor; the binding of IL-4 to unbounded receptor IL-4R is resumed by the term $\theta_{4}I_{4}n_{4}$ **.** The terms in Eq. (5) are identical to the terms expressed in Eq. (3) which is modeled with respect to IL-2 and IL-2R. Likewise, except the additional term $\kappa N_{4}n_{2}$ in Eq. (4), all the terms involved in Eq. (4) are identical to the terms in Eq. (2) with respect to IL-2 and IL-2R where $\kappa N_{4}n_{2}$ models the phenomenon of down-regulation of unbounded IL-2R by IL4 binding. The parameter κ defines the downregulation of IL-2R by IL-4.

### Dynamics of T Cells Activation and Proliferation

CD4+ T cells are a key part of the adaptive immune system. They differentiate into different phenotypes to carry out different functions. With the purpose of understanding the immune system as an integrated whole, Wertheim *et al.* present a new multi-approach and multi-scale modeling framework that can be used to model diverse immune responses at molecular, cellular, and systemic scales (Wertheim et al., 2021). In this study, cell population dynamics and systemic cytokine concentrations were described by ODEs (Wertheim et al., 2021). Excitingly, their model demonstrates the dynamics of CD4+ T cells in response to influenza infections in different cytokine milieus, considering heterogeneous populations of T helper cells and 11 cytokines in three spatial compartments (an infection site or target organ, lymphoid tissues, and a circulatory system). Furthermore, studies on the interplay among extracellular cytokines and the differentiation of T cells provide new insights into the plasticity of CD+ T cell differentiation and the generation of T cell phenotypes (Puniya et al., 2018). They model cellular dynamics in terms of concentration of cytokines which in turn control the differentiation of CD4+T cell. Similarly, MMQE uses a multiscale and multicellular model to assess immune response of T cells in terms of different activation protocols of the immune system in addition to the downregulation effect of IL-4 on IL-2 in the presence of antigens.

By default, T-cell antigen receptor (TCR) signaling is essential for activation, proliferation, and effector function of T cells. To this end, much attention has been focused on determining the minimum signaling requirements for TCR driven proliferation (Au-Yeung et al., 2017). Produced primarily by activated T cells, IL-2 enhances activation of T cells as well as promotes the differentiation of T cells into effector T cells and memory T cells (Liao et al., 2011). IL-2 serves dual opposing functions; it potently amplifies proliferative responses of effector T (Teff) and natural killer (NK) cells, while regulating immune homeostasis by driving regulatory T (Treg) cell proliferation, differentiation, and function (Abbas et al., 2018) and both axes have been leveraged to treat human diseases (Ghelani et al., 2020). Consequently, neither naive CD4 nor naive CD8 T cells are responsive to low doses of IL-2 (Au-Yeung et al., 2017), they both require a threshold of IL-2 signal that would selectively induce their proliferation after activation (Au-Yeung et al., 2014).

We propose a non-spatial and continuous time ODE to describe T-cell responses with regards to 1) the activation with the APCs, 2) the disappearance, and 3) the expansion of activated T cells during the presence of pathogens. The differentiation of helper T cell is controlled by the APCs Agents, which translates information about the microbial threat to the T cells. However, the activation of these helper T cells is performed by the classical APCs (DC, and B cells) which present MHC II peptide complex (Amsen et al., 2004). The main role of helper T cells is to mediate both the humoral and cellular immunity response by direct receptor binding or the releasing of specific cytokines that boost the immune response (Bianca et al., 2012). Since there is possibility that the helper T cell get activated independently by DC and B cells or both simultaneously, we assigned a variable ( Λ ) to the classic APCs defining the activation protocols controlled by Agents DC and B cells in the MMQE.

The mathematical model describing the dynamics of helper T cells would be a system of nonlinear first order ODEs that capture the additional time dependent change of the different levels of T cells representing linear independent factors depending on: 1) the distribution of the agent of activation, 2) the level of the agent of proliferation (cytokines), and 3) the death of T cells. Thus, it is expressed as:

$$\frac{dT_{t}}{\mathrm{dt}}=\phi_{\mathrm{Th}}\Lambda_{t}+\sigma_{2}\left(\frac{N_{2}}{N_{2}+\tau_{2}}\right)T_{t}-\delta_{\mathrm{Th}}T_{t}$$ (6)

where the term $\phi_{\mathrm{Th}}\Lambda_{t}$ performs the estimation of the activated helper T cell with rate parameter $\phi_{\mathrm{Th}}$ and the dependent activation variable $\Lambda_{t}$ which varies in terms of the activation protocol controlled by DC and B cells. The term $\sigma_{2}\left(\frac{N_{2}}{N_{2}+\tau_{2}}\right)T_{t}$ in Eq. (6) describes the stimulation of T helper cells by the cytokine IL-2 with level $N_{2}$ , threshold $\tau_{2}$ and the stimulation rate $\sigma_{2}$ **.** It is worth noting that the threshold represents the level of immunogenic stimulation required to elicit an immune response, determined by the sum of the negative regulatory mechanisms that work at all levels of the immune system (Guram et al., 2019). Stimulation of the immune system to any level below this threshold may still activate individual cells of the immune system but is insufficient to overcome various regulatory mechanisms and mount an effective systemic immune response. The T cell death factor is modeled by the term **−** $\delta_{\mathrm{Th}}T_{t}$ with death rate $\delta_{\mathrm{Th}}$ .

### Dynamics of B Cells Activation, Differentiation, and Proliferation

Germinal centers (GCs) are specialized compartments within the secondary lymphoid organs, where B cells proliferate, differentiate, and mutate their antibody genes (Martínez et al., 2012). Upon exit from the GC, B cells terminally differentiate into plasma cells or memory B cells (Thomas et al., 2019). The GC is divided into two functionally distinct zones, a dark zone, where B cells proliferate, and a light zone, characterized by the presence of follicular dendritic cells and bound antigen and which is associated with B cell selection (Camacho et al., 1998; Victora and Nussenzweig, 2012). Investigating the release of B cells from the GC (Meyer-Hermann et al., 2012) has identified that antigen-retaining B cells differentiate to plasma cells and leave the GC through the dark zone. This is of great importance in Synthetic Biology since it will allow a better quantification of B cells exiting from GC which will provide a better prediction on its differentiation into plasma cells. Additionally, the modeling of B cell maturation enables the characterization of the evolutionary process and competition at the heart of the GC dynamics (Pélissier et al., 2020). It shows that during an immune response one clone (or a small number of clones) of B cells is preferentially selected and proliferated, apparently at random, from a heterogeneous population of cells capable of responding to the given antigen (Williamson et al., 1976; Pélissier et al., 2020). Based on T cell-dependent activation of B cells (Liao et al., 2017; Cox and Brokstad, 2020), we aim to evaluate the dynamic proliferation of B cells in terms of 1) different APCs activating the immune systems and 2) the effect of the interaction between IL-2 and IL-4.

We model the dynamics of B cell population immune response as a function of B-cell activation, disappearance, or differentiation into more antibodies. With regards to their activation, B lymphocytes use B cell receptors (BCRs) to sense the physical features of the antigens so B cell receptor (BCR) signaling is pivotal for optimal B cell activation and differentiation (Guo and Rothstein, 2013; Wan et al., 2015). Hence, BCR interaction with an antigen should overcome the “survival” threshold to achieve full B cell activation (Avalos et al., 2014). In this regards, IL-4 was shown to provide survival or threshold signals to BCR for the activation and proliferation of B cells (Illera et al., 1993; Chung et al., 2002). This makes IL-4 to play an essential role in the activation of mature B cells as a cofactor for CD40L, and antigen stimulation to induce B cell differentiation, proliferation, and antibody secretion (Granato et al., 2014). Furthermore, the B cells begin to proliferate rapidly in the presence of the proper cytokines (IL-2 and IL-4), at their threshold levels (Feldkamp and Carey, 1996).

B cells are well known for their ability to differentiate into antibody secreting plasma cells in response to foreign antigens or pathogens (Lund, 2008). However, recent studies suggested that B cells also appear to regulate both protective and pathologic immune responses by an antibody independent mechanism (Hurdayal et al., 2017). Hence, after B cells get activated by IL-4, in a first stage, they can act as specialized antigen presenting cells, by recognizing pathogens through their specialized receptors and can then present peptide sequences to helper T cells. Consequently, a successful interaction with helper T cells, results in differentiation of B cells into plasma B cells, which release more of the same stimulating antibodies. Therefore, modeling the dynamics of B cell immune response should include frameworks that investigate on 1) the activation of B cells by the cytokine IL-4, 2) the proliferation or stimulation of activated B cells, and 3) the differentiation and death of mature B cells (Bianca et al., 2012; Figueredo et al., 2013). Considering these three points, the model’s equation illustrate the dynamics between B cell ( $B_{t}$ ), T cells ( $T_{t}$ ) and the cytokines IL-2 ( $N_{2}$ ), and IL-4 ( $N_{4}$ ) described by the following differential equation:

$$\frac{dB_{t}}{\mathrm{dt}}=\phi_{B}\left(\frac{N_{4}}{N_{4}+\tau_{4}}\right)T_{t}+\sigma_{2}\left(\frac{N_{2}}{N_{2}+\tau_{2}}\right)B_{t}-\left(\phi_{p}+\delta_{B}\right)B_{t}$$ (7)

where the term $\phi_{B}\left(\frac{N_{4}}{N_{4}+\tau_{4}}\right)$ performs the activation and proliferation of B cells with respect to IL-4 where $N_{4}$ stands for the bounded level of IL-4 to IL-4R. $\phi_{B}$ and $\tau_{4}$ represent the activation rate of IL-4 and the binding threshold of IL-4 respectively. The term $\sigma_{2}\left(\frac{N_{2}}{N_{2}+\tau_{2}}\right)$ describes the proliferation of B cells by duplication due to IL-2 with $N_{2}$ defining the bounded level of IL-2 to IL-2R and, $\sigma_{2}$ and $\tau_{2}$ represents the duplication rate and the binding threshold of IL-2 respectively. The term $-\left(\phi_{p}+\delta_{B}\right)$ emphasizes the differentiation and the death of the mature B cells ( $B_{t}$ ) where $\phi_{p}$ and $\delta_{B}$ represent the differentiation and death rate respectively of mature B cells.

Besides being able to recognize antigens such as APC, B cells can be differentiated into a plasma B-cell, whose specialized job is to mass-produce the antibodies that match the activating invader (antigen) and provide immune protection for the human body (Hoffman et al., 2016). Thus, each plasma B-cell makes antibodies unique to only one antigen. Hence, B cells leave the germinal centers at rate $\phi_{p}$ to become plasma B cells ( $P_{t}$ ) through the ODE (Erwin and Ciupe, 2017):

$$\frac{dP_{t}}{\mathrm{dt}}=\phi_{p}B_{t}$$ (8)

B cells maintain immune tolerance and suppress a wrong or excessive immune response to avoid damage to the body by controlling their rate of differentiation, proliferation, and death.

### Dynamic of Activation Variable for Different Activation Protocols

Classical APCs including DC and B cells are a heterogeneous group of immune cells that mediate the cellular immune response by processing and presenting antigens for recognition by certain lymphocytes such as T cells. Therefore, we illustrate the dynamic of the dependent activation variable $\Lambda_{t}$ in Eq. (6) in terms of the dynamic proliferation of agents DC and B cells as follow.

• In the case where DC Agent makes helper T cells recognize the antigens for its activation (Figure 1A) then, the distribution of the activation variable ( $\Lambda_{t}$ **)** will be defined in respect of the differentiation of agent DC as:

$$\frac{d\Lambda_{t}}{\mathrm{dt}}=\phi_{\mathrm{DC}}A_{\mathrm{ag}}-\delta_{\mathrm{DC}}\Lambda_{t}$$ (9)

where $\phi_{\mathrm{DC}}$ and $\delta_{\mathrm{DC}}$ serve as the activation and the death rates of DC respectively, and $A_{\mathrm{ag}}$ defines the variable allocated to agent of antigens.

• In the case B cells play the role of APC in activating helper T cells (Figure 1B), the distribution of the activation variable ( $\Lambda_{t}$ ) will depend on the dynamical differentiation of B cells in the circulation proposed in Eq. (7). It follows then:

$$\frac{d\Lambda_{t}}{\mathrm{dt}}=\phi_{B}\left(\frac{N_{4}}{N_{4}+\tau_{4}}\right)T_{t}+\;\sigma_{2}\left(\frac{N_{2}}{N_{2}+\tau_{2}}\right)\Lambda_{t}\;-\left(\phi_{p}+\delta_{B}\right)\Lambda_{t}$$ (10)

• After long exposure of T cells in the pathogen environment or for a high number of antigens in the immune system, DC could miss the identification of certain antigens which would be caught by B cells in the circulation. Accordingly, Agents DC and B cells would play a joint role of facilitating antigen recognition by T cell for its activation (Figure 1C).

## Results

### Numerical Evaluation

We first obtain estimates for the parameter values and initial conditions for our model from published *in-vitro* or *in-vivo* experimental research. All the parameters involved in (Eqs 2–10) are summarized in Table 2. However, by referring to the initial conditions, we provide more details on some parameters such as: the initial number of naïve T cells and B cells, the death rates of T and B cells, the production rate of the plasma B cells, the mean and variance of the lognormal distribution function of the pathogens. The microscopic states of antigens are set to the lognormal distribution with mean $\mu_{ag}=0.075$ and variance $V_{ag}=1$ . This will present a continuous skewed variation of pathogens and will enable the progressive evaluation of the proliferation of lymphocytes as immune responses. The population of T cells in the body is naturally divided into clonotypes which represents the set of cells that have identical T-cell receptors (Lythe and Molina-París, 2018). The total number of cells, such as naïve CD4+ T cells, are large enough to be modeled by ordinary differential equations using appropriate starting points. All naïve CD4+ T cells are about $10^{7}$ in an adult mouse or about $10^{11}$ in an adult human (den Braber et al., 2012). Accordingly, we set the initial number of naive CD4+T cells ( $Th_{0}$ ) which is recruited by the antigens ( $A_{ag}$ ) as $Th_{0}=805$ cells per ml. The death rates of all CD4+T cells are equal to $\delta_{Th}=0.01\;\left(s^{-1}\right)$ such that the activation rate of helper T cells is defined as: $\phi_{Th}=Th_{0}\times \delta_{Th}$ . The adaptive immune response is slow to develop on first exposure to a new pathogen, as specific clones of B cells have to become activated and expand; it can therefore take time before the response is effective (Roberts et al., 2002). Here, we assume that at the initial time when the body is exposed to the pathogens, T cells receive the first signal before communicating with B cells. Therefore, at the initial time, we consider a negligible number of B cells (approximately 100 cells per ml) comparing it to the initial number of T cells. The death rate of mature B cells ( $\delta_{B}$ ), the activation rate of B cells ( $\phi_{4}$ ) and the production rate of plasma B cells are defined as: $\delta_{B}=0.01\;\left(s^{-1}\right)$ , $\phi_{B}=1\;\left(s^{-1}\right)$ and $\phi_{p}=3\;\left(s^{-1}\right)$ respectively. Moreover, due to the fact that the regulation of cytokines is necessary in mediating a pathogen invasion response and only limited information is available on the timing of cytokine production and decay (Thakar et al., 2007), we assume that the regulatory dynamics of all the three lymphocytes (helper T cells, B cells and plasma B cells) are independent to each other.

**TABLE 2:** Parameter values Parameters involved in Eqs 2–10 describing effects of IL-2 and IL-4 on the proliferation of T, B, and Plasma B cells in response to the invasion of pathogens. All values present in this table are collected from published results of *in-vitro* or *in-vivo* experimental research.

Parameter	Symbol	Value	References
DC activation rate	$\phi_{DC}$	0.07 ${\left(s\right)}^{-1}$	Bianca et al. (2012)
DC death rate	$\delta_{DC}$	$3.97e^{-2}{\left(s\right)}^{-1}$	Zehn et al. (2004)
Average production rate of unbounded IL-4	$\phi_{4}$	7,080 ${\left(s\right)}^{-1}$	Lowenthal et al. (1988)
Variance of production rate of unbounded IL-4	$V_{4}$	1	Burke et al. (1997)
Rate of IL-4 binding to IL-4R	$\theta_{4}$	$3.8e^{7}{\left(M.s\right)}^{-1}$	Lowenthal et al. (1988)
Dissociation rate of IL-4 from IL-4R	$\varphi_{4}$	$18.6e^{-4}{\left(s\right)}^{-1}$	Lowenthal et al. (1988)
Unbounded IL-4R internalization rate	$\gamma_{4}$	$0.35e^{-4}{\left(s\right)}^{-1}$	Lowenthal et al. (1988)
Bounded IL-4R internalization rate	$\delta_{4}$	$5.27e^{-4}{\left(s\right)}^{-1}$	Lowenthal et al. (1988)
Average production rate of unbounded IL-2	$\phi_{2}$	1,698 ${\left(s\right)}^{-1}$	Fernandez-Botran et al. (1989)
Variance of production rate of unbounded IL-2	$V_{2}$	1	Fernandez-Botran et al. (1989)
Rate of IL-2 binding to IL-2	$\theta_{2}$	$23.46e^{7}{\left(M.s\right)}^{-1}$	Hurtley and Helenius (1989)
Dissociation rate of IL-2 from IL-2R	$\varphi_{2}$	$2.09e^{4}{\left(s\right)}^{-1}$	Hurtley and Helenius (1989)
Unbounded IL-2R internalization rate	$\gamma_{2}$	$1.4e^{-4}{\left(s\right)}^{-1}$	Duprez et al. (1988)
Bounded IL-2R internalization rate	$\delta_{2}$	$5.62e^{-4}{\left(s\right)}^{-1}$	Duprez et al. (1988)
Parameter for down-regulation of IL-2R by IL-4	κ	$8.88e^{-8}{\left(s\right)}^{-1}$	Burke et al. (1997)
Stimulation rate of IL-2	$\sigma_{2}$	$9.10e^{-3}{\left(s\right)}^{-1}$	Bianca et al. (2012)
Threshold level of IL-2	$\tau_{2}$	$2e^{4}{\left(s\right)}^{-1}$	Burke et al. (1997)
Binding threshold of IL-4 for B cell activation	$\tau_{4}$	$18e^{4}{\left(s\right)}^{-1}$	Burke et al. (1997)
Differentiation rate of mature B cell	$\delta_{d}$	$0.16e^{4}{\left(s\right)}^{-1}$	Muñoz-Carrillo et al. (2019)

The model contains 19 parameters which have specific biological meanings. These parameters have been derived from literature, from measurements in different *in-vivo* experiments which is similar to other immune system studies (Bianca et al., 2012; Gao et al., 2016; Nickaeen et al., 2019). Therefore, to account for uncertainty in computational predictions, we simulated our model by taking account of error margins allowing parameters to vary in the range $\left[\text{Parameter}-{\text{σ}}_{p},\text{ Parameter}+{\text{σ}}_{p}\right]$ where ${\text{σ}}_{p}$ represents the estimated standard error corresponding to the 95% confidence interval for the model parameters. Varying parameter values in a suitable range while fixing the rest, represents the classical way to do sensitivity analysis (Bianca et al., 2012).

### Downregulation of IL-2 by IL-4

**FIGURE 3:**
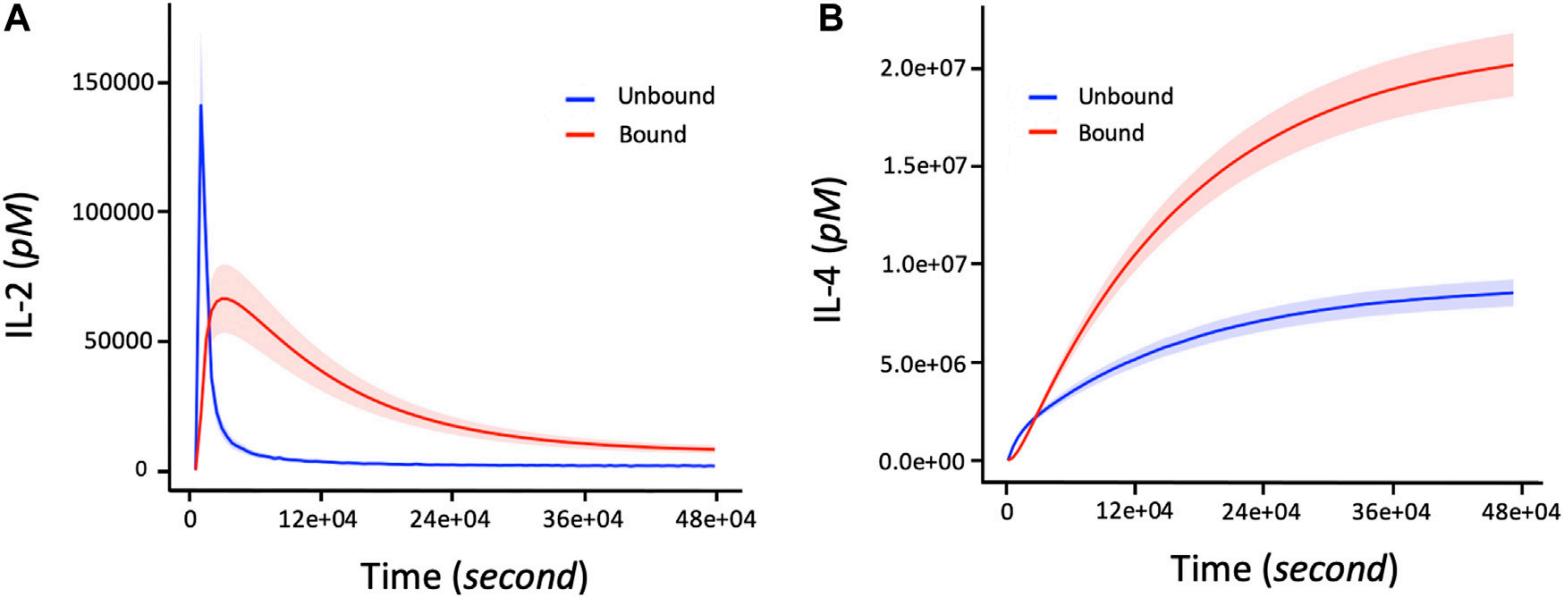
The number of bounded and unbounded IL-2 and IL-4 receptors driving the proliferation response of T cells and B cells in the immune system over time. The parameter values (in Eqs 2–5) used to obtain these curves are shown in Table 2. The initial corresponding concentration of IL-2 and IL-4 used are respectively 1.5 and 15pM. **(A)** Non-linear profile representing the dynamic changes of IL2 bounding to receptors. The bounded number of IL-2 correspond to the number of IL-2 involved in the proliferation of T cells and B cells during an immune response. **(B)** Increasing changes of the bounded number of IL-4 corresponds to the level of cytokine IL-4 connected to the activation and proliferation of B cells. The shaded areas of IL-2 and IL-4 represent the confidence intervals at 95% confidence level of parameter values of the model.

We first evaluate our model immune response resulting from the interactions between IL-2 and IL-4 ligand binding and their receptors. A rapid association rate of IL-2 binding to IL2-R occurs followed by a slower dissociation rate (Figure 3A) for both bounded and unbounded ligands. Specifically, the maximal rate of association for bounded ligands seems to occur after 15,000 s. A rapid maximal rate of dissociation of IL-2 from IL-2R occurs after 6,000 s for unbounded ligands. On the other hand, IL-4R binding shows a much slower association rate of IL-4 ligand as well as a longer delay of dissociation rate of IL-4 from IL-4R (Figure 3B). Quantitatively, the maximum rate of binding of IL-4 to IL-4R or unbinding rate of IL-4 from IL-4R can take a long time [defined in the range (36e+04, 48e+04) seconds] to complete. Note that the number of IL-4 bounded to the IL-4R is double the number of IL-4 unbounded from the IL-4R. Additionally, since the binding or unbinding mechanisms of IL-2 and IL-4 to/from IL-2R and IL-4R occurs simultaneously in response to the presence of the pathogens in the immune system, it is worth noting that the more the bounded number of IL-4 to IL-4R increases, the more the bounded number of IL-2 to IL-2R decreases (Figures 3A,B).

### The Immune Response Depends on Activation Agents and Joint Interplay of IL-2 and IL-4

Upon recognition of specific antigens via their T-cell receptor (TCR) in the context of appropriate co-stimulatory signals, T cells clonally expand and traffic to tissues, where they perform effector functions including direct killing of infected cells and secretion of cytokines coordinating local immune responses (Brenchley et al., 2002). Sustained B cell activation also leads to increase in B cell proliferation and differentiation. In response to activation signals, naive mature B cells proliferate and differentiate into effector cells. This is driven by integration of several infection-related signals, including binding of specific antigens to the B cell receptor (BCR) and pattern recognition receptor ligands (Ruprecht and Lanzavecchia, 2006; Pone et al., 2010).

**FIGURE 4:**
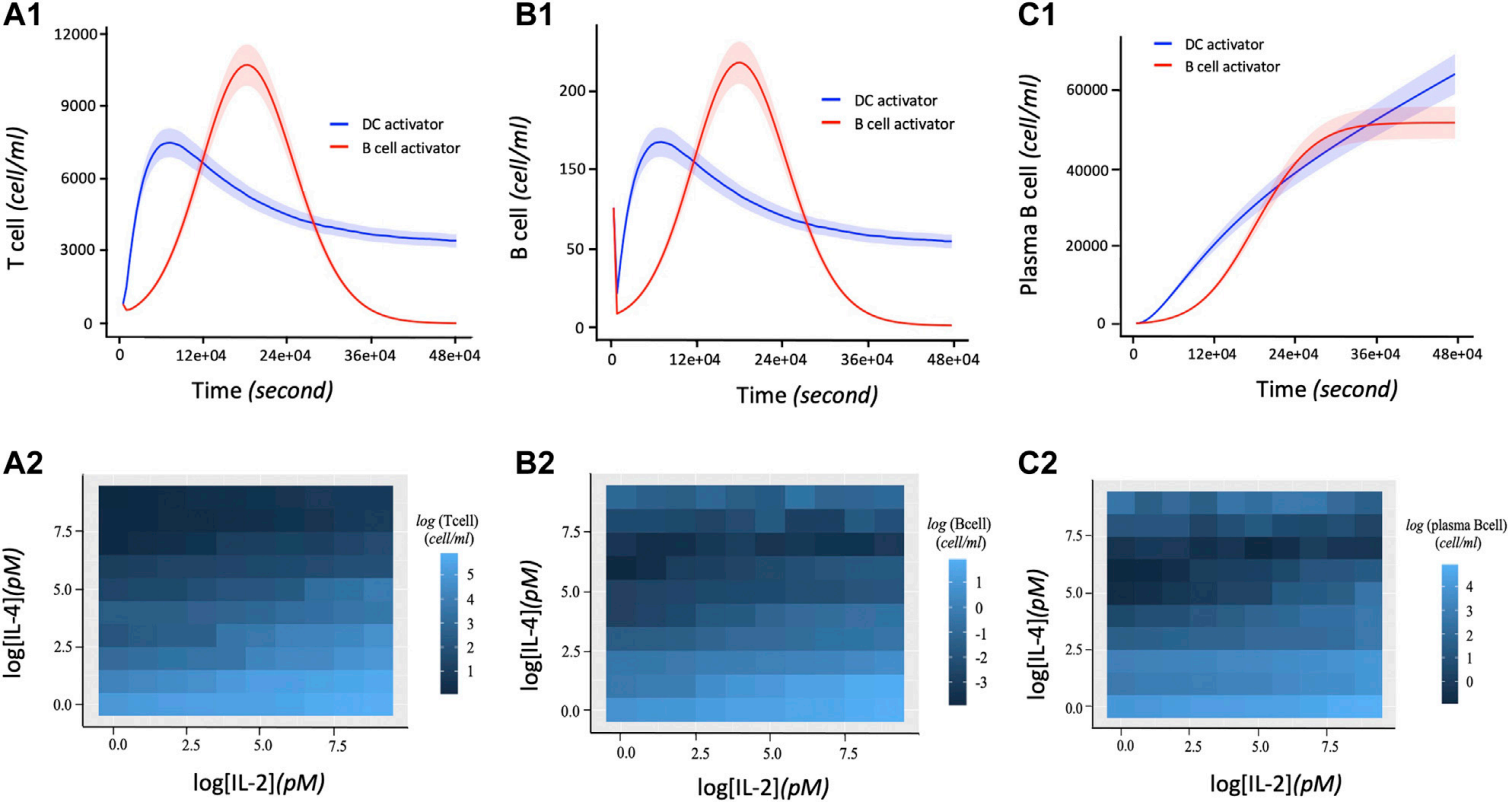
Dynamic profiles of lymphocyte immune response with respect to DC and B cell activation protocols. The parameter values (in Eqs 1–10) used to obtain these curves are shown in Table 2. 1.5 and 15pM concentration levels of IL-2 and IL-4 are used respectively. **(A1)** Number of T cells proliferation over time in terms of independent DC and B cells activators. **(B1)** Rate of B cell proliferation with regards to DC and B cell activation of the immune system. **(C1)** Dynamic proliferation of Plasma B cells with respect to the DC and B cell activations of the immune system. The shaded area of the levels of T cells, B cells and Plasma B cells in **(A1)**, **(B1)**, and **(C1)** respectively represent the 95% confidence interval of the model parameters. **(A2)** Heatmap describing the effect of the downregulation of IL-2 by IL-4 correlates with an increase in T cell proliferation. A high T cell proliferation is proportional to low concentration of IL-4 and high concentration of IL-2. **(B2)** Heatmap showing the co-expression patterns of IL-2 and IL-4 signaling and the B cell proliferation. Initially, high B cell proliferation is obtained with a high concentration of IL-2 and low concentration of IL-4. Due to the ability of IL-4 to co-stimulate B cell proliferation (Idzerda et al., 1990), an extreme high concentration of IL-4 presents a slight increase of B cells proliferation. **(C2)** Heatmap describing the joint effect of the downregulation of IL-2 by IL-4 on the dynamical proliferation of Plasma B cells. High Plasma B cell proliferation correlates more with low concentration of IL-4 and high concentration of IL-2. However, like B cell, it also displays a slight increase of its proliferation at high concentration of IL-4 considered as co-stimulator of B cell proliferation.

**FIGURE 5:**
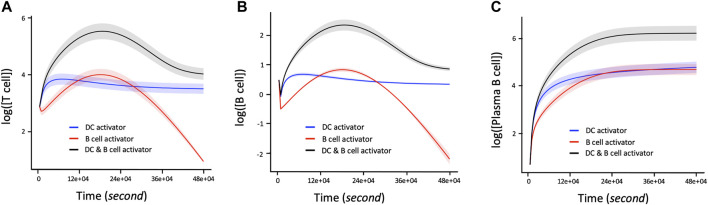
Time dependent Lymphocyte immune response with respect to three activation protocols: DC activation, B cell activation, joint activation of DC and B cells. The corresponding concentration of IL-2 and IL-4 used are 1.5 and 15pM respectively. **(A)** Log 10-time dependent changes in number of T cells in terms of DC activator, B cells activators, and the joint DC and B cells activations. **(B)** Log 10 of changes in number of B cells over time with regards to DC activator, B cell activator and the joint DC and B cell activator. **(C)** Rate of change of proliferation values in Log 10 scale corresponding to the number of Plasma B cells per unit time in seconds with respect to the DC activator, B cell activator, and the joint DC and B cells activator. The shaded area of the levels of T cells, B cells and Plasma B cells in **(A)**, **(B)**, and **(C)** respectively represent the confidence interval at 95% confidence level.

The proliferation of T and B cells increases to a maximal level followed by degradation regardless of the immune system activation agent (Figures 4A1, B1). However, the maximum number of T and B cells produced during their proliferation lifecycle depends on the activation agent. For example, B cell activating agents lead to a higher maximal level of T and B cells proliferation compared to DC agents. In addition, the proliferation of T cells and B cells immune response with respect to the B cell agent activator decreases from its maximum level to zero after about 390000s of proliferation. On the other hand, the proliferation of T and B cells based on DC agent activators reaches steady state during the same period. Conversely, the proliferation levels of plasma B cells (Figure 4C1) reach steady state earlier at 270000s when the activation mechanism is regulated by B cell agent. However, their proliferation rate keeps increasing with time when the immune response is activated by DC agents. In addition to regulating B cell growth and immunoglobulin secretion, IL-4 also affects the proliferation of lymphocytes. The proliferation level of T, B and Plasma B cells tightly depend on the interaction between cytokines IL-2 and IL-4. Hence, T cells, B cells, and Plasma B cells together display a high level of their immune response which is associated with a low concentration of IL-4 versus high concentration of IL-2 (Figures 4A2, B2, C2). In other words, the concentration of IL-4 decreases proportionally with increase in proliferation levels of T and B cells which automatically influences the growth of the Plasma B cells. However, the level of proliferation of T and B cells is highly influenced by the activation protocol defined by the joint activation with B cell and DC. Thus, T and B cells present a higher level of proliferation comparing it to the case where the immune system was activated independently by B cell or DC (Figures 5A,B). Similarly, the proliferation of plasma B cells reached a high level of steady state after 270000s of proliferation time (Figure 5C). Supplementary Figure S1–S3 provides additional plots of similar dynamic profiles as in Figure 3 and 4 of lymphocyte proliferation from different proportions of IL2-IL4 concentration levels.

We next calibrate the model by investigating the effects of simultaneously varying IL-2 and IL-4 concentration levels that are likely to influence the productivity growth of the lymphocytes during an immune response. Due to the permanent exposure of lymphocytes to pathogens in the immune system, our aim is to determine the rate of proliferation of lymphocytes during the immune response in relation to the abundance of cytokines IL-4 and IL-2 acting jointly on individual cells. We investigate the marginal effects of eight different intervals of concentrations of IL-2 and IL-4 in *pM* as follows: C0=[0−0.75] , C1=[0.76−1.5] , C2=[1.51− 2.25] , C3=[2.26−3] , C4=[3.01−3.75] , C5=[3.76−4.5] , C6=[4.51−5.25] , C7=[5.26−6] . From each interval, we 1) generate uniformly 50 samples of concentration of IL-2 and IL-4 to compute the proliferation of lymphocytes during immune response based on (Eqs 6–10, Eq. 2) and identify the maximal level of each lymphocyte produced during their proliferation. Accordingly, we collect in total 50 data points of maximal proliferation levels of each of the lymphocytes associated with each interval.

Denoting $y_{1},...,y_{k}$ for k=50 as the maximal proliferation levels of lymphocyte collected in each range of cytokine concentration range $R\;=\left[{\left[cytokine\right]}_{min},{\left[cytokine\right]}_{max}\;\right]$ , we define the rate of proliferation growth ( $\gamma_{p}$ ) as:

$$\gamma_{p}=\frac{\mathrm{mean}\left(y_{1},\cdots ,y_{k}\right)}{\mathrm{sum}\left(y_{1},\cdots ,y_{k}\right)}\times 100$$

**FIGURE 6:**
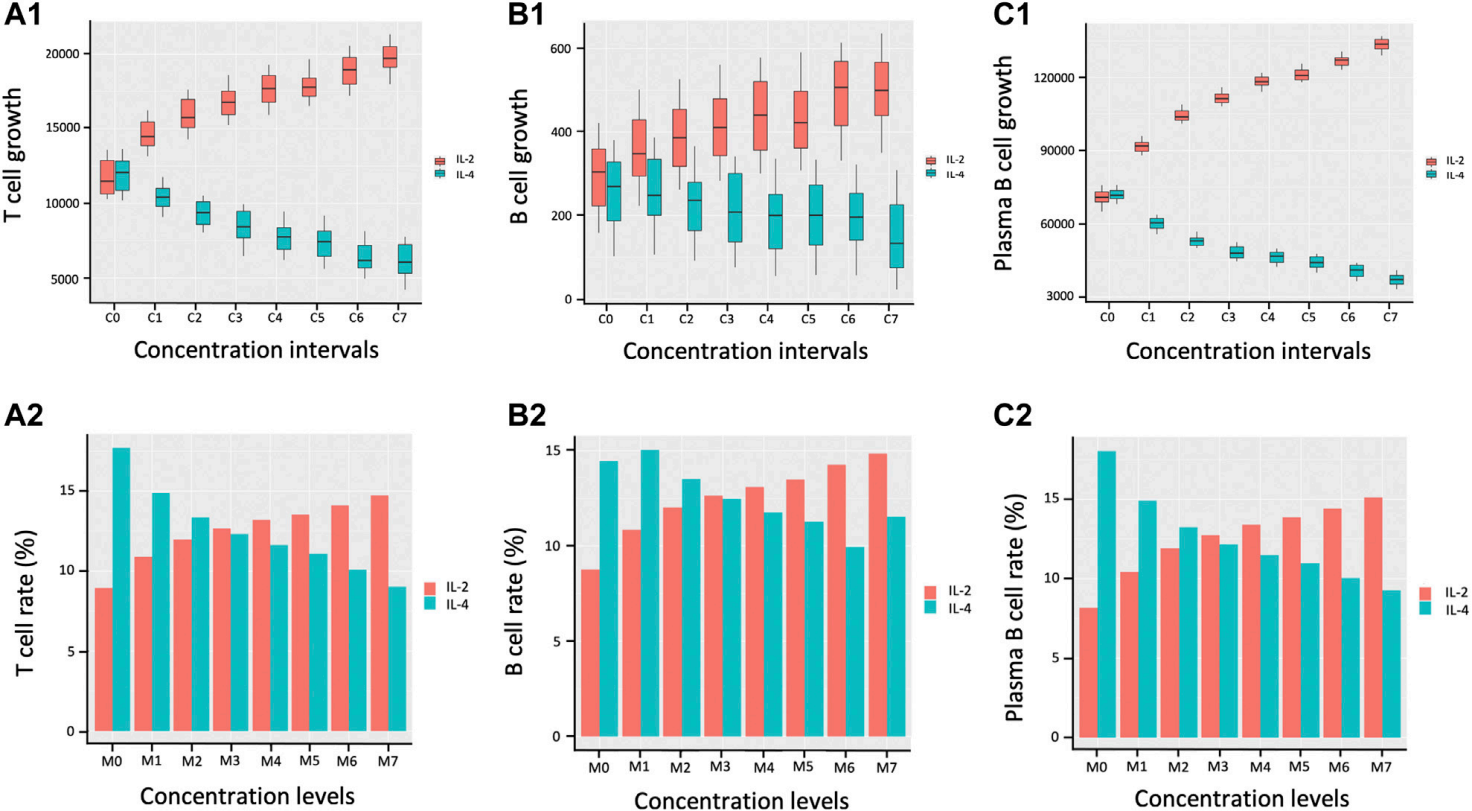
Proliferation growth and percentage growth rate of lymphocytes during immune response. C0, C1, C2, C3, C4, C5, C6, and C7 represent eight matched concentration intervals of IL-2 and IL-4 in *pM* respectively: C0=[0−0.75] , C1=[0.76−1.5] , C2=[1.51− 2.25] , C3=[2.26−3] , C4=[3.01−3.75] , C5=[3.76−4.5] , C6=[4.51−5.25] , C7=[5.26−6] . For each range of concentration of cytokines IL-2 and IL-4, 50 random samples are generated uniformly. The distribution and maximal levels of helper T cells, B cells and plasma B cells produced during their proliferation time are evaluated for each concentration sample of cytokines. The parameter values used to obtain these plots are shown in Table 2. In **(A1)**, **(B1)**, and **(C1)**, the lymphocyte proliferation growth displays a decrease in growth average patterns with decrease in the concentration levels of IL-2 with a corresponding rise of the concentration of IL-4. M0, M1, M2, M3, M4, M5, M6, and M7 represent eight matched average concentration of intervals C0, C1, C2, C3, C4, C5, C6, and C7 for IL-2 and IL-4 in *pM* respectively: M0=0.37 , M1=0.74 , M2=1.11 , M3=1.48 , M4=1.85 , M5=2.22 , M6=2.6 , M7=3 . **(A2)**, **(B2)**, and **(C2)**, present the rate of proliferation growth of all the three lymphocytes which is approximately identical to the growth patterns regardless of the varying concentration of IL-2 and IL-4. This leads to the conclusion that the interplay between IL-2 and IL-4 has identical effects on T, B, and Plasma B cell proliferation modality.

Figure 6 displays the bar plots representing both lymphocyte proliferation growth in terms of number of cells as well as the associated rates of proliferation growth across various cytokine concentration intervals. The proliferation of T cells, B cells, and plasma B cells is left skewed with decrease in the concentration levels of IL-2 and right skewed with increase in IL-4 concentration indicating opposing trends with plasma B cells showing the largest difference.

### Model Validation

Our computational model aims to explain biological phenomenon of T cells activation followed by the proliferation of T, B and plasma B cells, as well as the antagonistic effects of IL-2 and IL-4 on lymphocyte proliferation in the presence of antigens. CD4+ T cell functions include activating other immune cells, releasing cytokines, and helping B cells to produce antibodies as well as differentiate into plasma B cells. Due to CD4+ T cell’s ability to shape, activate and regulate the adaptive immune response (Henninger et al., 2010; Swain et al., 2012), we validate our model by targeting T cells as a variable of interest based on their relative importance in immune response (Henninger et al., 2010). We analyze data from two different experimental studies (Morris et al., 2000; Ahmadi et al., 2008) to help validate 1) the comparison of CD4+ T cell proliferation with the activation of the naïve T cells initiated by different APCs: activated B cells, and mature DC (Supplementary Figure S4); 2) the influence of the antagonistic effect of cytokines IL-2 and IL-4 on the proliferation of CD4+ T cells (Supplementary Figure S5).

**FIGURE 7:**
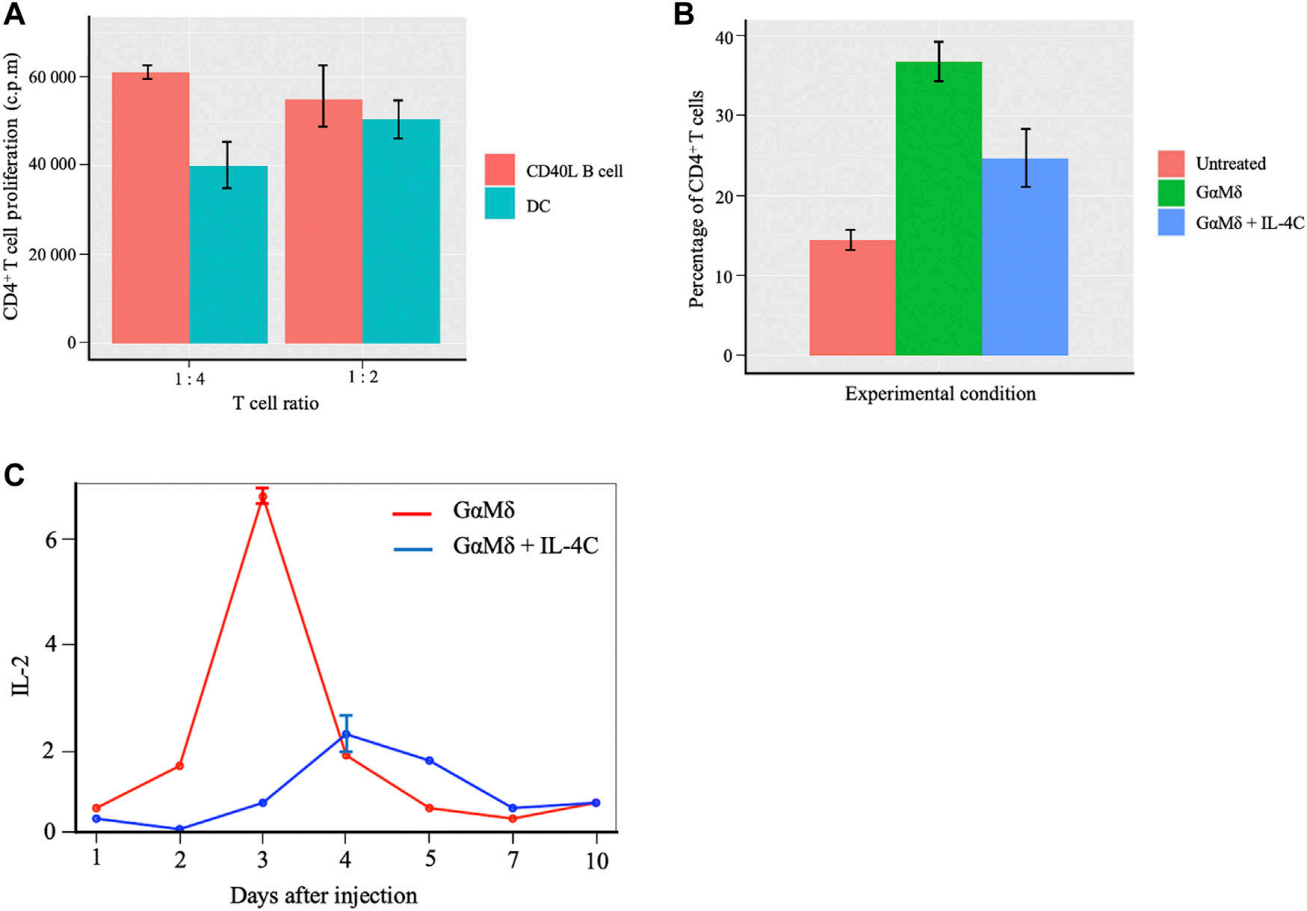
Proliferation of CD4+ T cells with B cells and DCs activation of the naïve T cells and effects of cytokines IL-2 and IL-4 on T cells proliferation. **(A)** Barplots showing comparable effects of Murine CD40L-activated B cells to mature dendritic cells (DC) in peptide presentation to CD4+ T-cell clones. Data obtained from independent cultures of APC are presented as means ± SE. The CD4+ T-cell clones were cultured in the presence of APC. **(B)** Barplots showing differences in percentage change of CD4+ T cells between three conditions; BALB/c mice were left untreated (Untreated), BALB/c mice induced with IL-4C signaling and injected with antigen and mice only injected with antigen alone. IL-4C inhibits GαMδ induction of CD25 expression by CD4+ T cells. Representative barplots are shown for the fluorescence of CD25 on CD4+ T cells which represent means and SE for percentages of CD4+ cells that express CD25. A higher CD4+ T cell production in the presence and absence of IL-4C stimulation is observed compared to the untreated condition. **(C)** Curves showing the inhibition of IL-4C gene expression of IL-2 under two conditions. In the first scenario, BALB/c mice were injected with 800 μg of GαMδ on day 0 (GαMδ treated). In the second condition, BALB/c mice were injected with 800 μg of GαMδ + IL-4C (5 μg of IL-4 + 30 μg of anti-IL-4 mAb) on day 0 (GαMδ + IL-4C treated). Mice were sacrificed 1, 2, 3, 4, 5, 7, or 10 days after injection, and splenic cytokine gene was studied. IL-4C inhibition of GαMδ-induced IL-2 expression dynamics corresponds to a reduction of CD4+T cells or proliferation levels.

Figure 7A summarizes a direct head-to-head comparison of proliferation of CD4+ T cells between CD40L-activated B cells and mature DCs with regards to presenting antigens to the naïve T cells for activation (Supplementary Figure S2 for more details on the first study). It is worth noting that CD40 ligand (CD40L) is the critical membrane-expressed molecule responsible for T cell dependent B-cell activation denoted as CD40L-activated B cells serving as effective antigen APC. These data indicate that primary CD40L expanded B cells are efficient presenters of antigens to T cells and characterized CD40L-activated B cells as more influential APC compared to mature DCs. Figures 7B,C (Supplementary Figure S3), show differences in percentage change of CD4+ T cells and IL-2 signaling expression respectively between joint IL-4C signaling and antigen presence, and only antigen production. We observe a higher CD4+ T cell production in the absence of IL-4C stimulation (Figure 7B). The higher production of antibody by B-cells recognized by the increased presence of mouse antigen (GαMδ) resulted in the largest increase in the level of CD4+ T cells compared to the untreated case (case with no GαMδ). However, the treatment with IL-4C inhibited GαMδ-induced IL-2 response (Figure 7C) systematically shows a reduction of CD4+ T cells and proliferation levels since IL-2 is the cytokine growth factor of CD4+ T cells (Figure 7B). The above results provide evidence of the antagonistic effect of IL-4 on IL-2 by decreasing not only CD4+ T cell activation but also the T cell production of IL-2.

### Code Availability

We use a hybrid ODE models with a stochastic component called MMQE to capture the variability in dynamic interaction effects resulting from communication between extracellular and intracellular dependent agents (Figure 2) within an immune response environment. Our model combines agents using network-based rules within an ODE system with specific initial conditions presented in the subsection of *Numerical Evaluation*. We implemented the given ODEs and estimate parameters analytically using MATLAB (Macal and North, 2005; Cilfone et al., 2015; Martin and Schlüter, 2015; Hunter et al., 2018). The codes used in this study were written using MATLAB version MATLAB_R2021a and is available at https://github.com/atiteyk2/Downregulation_Immunology.git.

## Discussion

In this study, we investigate the joint effects of IL-2 and IL-4 stimulation and the heterogeneous lymphocyte proliferation changes during an immune response using a hybrid ODE model with a stochastic component framework describing the microscopic state of antigen invasion. We mechanistically model how the immune response to foreign invasion is dependent on the dynamic activation of lymphocyte agents, the joint interplay of IL-2 and IL-4 signaling, and the activation agent for the immune response. Both cytokines IL-2 and IL-4 are major mediators of adaptive immune response. IL-2 plays a crucial role maintaining peripheral self-tolerance by having both immuno-stimulatory and immuno-regulatory functions. It acts primarily as a T cell growth factor, essential for the proliferation and survival of T cells as well as the generation of effector and memory T cells (Kobayashi et al., 2014). IL-2 ligand binds dynamically and specifically to various forms of receptors including IL-2Rα (CD25), IL-2Rβ (CD122), IL-2Rγ (CD132) (Malek and Castro, 2010; Liao et al., 2013; Karakus et al., 2020) on the surfaces of different immune cell types primarily T cells, DCs and natural killer cells. For example, free IL-2 binds first to IL2-Rα expressed on the surface of activated DCs for trans presentation to neighboring cells including antigen-specific naïve T cells and NK cells that express both IL-2Rβ and IL-2Rγ chains (Kobayashi et al., 2014). This trans presentation of IL-2 has been shown to facilitate initial high-affinity IL-2 signaling and early immune response (Liao et al., 2013). Additionally, Wuest et al. (2011) showed that secretion of IL-2 by a mature DC towards the DC-T cell interface lends IL-2Rα (CD25) to prime transitioning T cells to facilitate early IL-2 signaling, which is crucial for subsequent T cell expansion and development of antigen-specific effectors. This justifies the observed early rise in cytokine signaling followed by a sudden decrease of the level of bounded and unbounded number of IL-2 receptors during an immune response (Figure 3A).

B cells generally receive help from cognate helper T cells and differentiate into plasma B cells that secrete large amounts of antibodies (LeBien and Tedder, 2008; Corcoran and Tarlinton, 2016). IL-4 is a cytokine with pleiotropic activity in the immune system whose stimulation activates B-cells and the differentiation of B cells into plasma cells. Specifically, IL-4 induces B-cell class switching to IgE, and MHC class II production (Oliver et al., 1985; Nelms et al., 1999) and also affects T cell proliferation. For example, *in vitro* experiments have shown that IL-4 induces the differentiation of naive CD4+ T cells into Th2 cells, which are characterized by their capacity to secrete the cytokines IL-4, IL-5 and IL-10 upon activation (Smiley et al., 1997). The multifunctionality of IL-4 (Luzina et al., 2012) in the immune system justifies the continuous increase in the level of IL-4 signaling during the duration of the pathogen in the immune system displayed in Figure 3B.

Furthermore, IL-4 has been reported to interfere with certain IL-2-mediated responses (Barcena et al., 1991) by counteracting several direct effects of IL-2. This study has consistently shown that the increase of the concentration of IL-4 in the immune system, reduces the effect of IL-2 in stimulating the proliferation of targeted lymphocytes. A possible explanation is that IL-4 induces the differentiation of naive CD4+ T cells into Th2 cells, which are characterized by their capacity to secrete the cytokines IL-4, IL-5 and IL-10 upon activation, while simultaneously inhibiting the generation of Th1 cells, which secrete IL-2 and interferon gamma (IFNγ) (Ratcliffe, 2016). Furthermore, IL-4 profoundly suppresses the number of high affinity binding sites for IL-2 on *in vitro* activated B cells (Galanaud et al., 1990), while concomitantly enhancing the specific response of normal B-cells. These independent findings jointly explain the observed interplay between the different lymphocyte proliferation growth rates and the antagonistic effects of IL-2 and IL-4 signaling in our study.

Cell-cell communication between targeted lymphocytes also drives adaptive immunity. Successful *in vitro* and *in vivo* experiments have shown that in the absence of B cells, T cells responses are greatly reduced (Rivera et al., 2001). B cells are regarded as professional antigen-presenting cells (APC) despite their primary role in humoral immunity (Chen and Jensen, 2008). In the last decade, investigations on B cell response have shown that although B cells are poor at presenting antigens via nonspecific uptakes, they capture and internalize cognate antigens that bound to their B cell receptors and present them very efficiently to helper T cells (Pierce et al., 1988; Lanzavecchia, 1990). Therefore, one can conclude that B cells could be the most efficient APC on a per cell basis for a particular antigen (Perera et al., 2013). Figure 4A1 and Figure 4B1 showing the dynamic changes in T and B cells proliferation for different activation agents justify that T cells activation by B cell population (B agent) produces a higher level of proliferation of T and B cell immune response compared to the case when DC activates the immune response (See Supplementary Figure S1). We hypothesis that DC and B Cells will simultaneously activate T Cells in the presence of a large numbers of antigen of the similar types. In this case, the results of our study display a fulgurant increase in antibody production when multiple types of the lymphocytes are involved.

We acknowledge the use of published independent studies to validate our work instead of a well-controlled experimental animal study. Also, a small number of biological species (immune cell types and cytokines) are used to define the complex immune response model in this study. However, the similar results of our study with other experimental investigations including the calibration analysis of model parameters provide some form of empirical validation and robustness of the model which can cost effectively be used in the field of synthetic biology for cells and cytokine productions to improve treatment for infectious diseases such as COVID-19 and cancer. For example, IL-2 is the only drug approved in the United States for the treatment of metastatic Renal cell carcinoma (RCC) and metastatic melanoma (Atkins et al., 1999; McDermott and Atkins, 2006). It can activate and stimulate the growth of immune cells, most importantly T-cells and Natural Killer cells, both of which can destroy cancer cells or many other infection disease pathogens directly with the combination of B cells to jointly slow disease progression and/or manage symptoms. The former can be controlled through using our proliferation response model of IL-2 and IL-4 signaling accompanied with using the B cells as activator of T-cells or the joint activation of T cells by DC and B cells. The latter toxicity concern can be controlled by estimating different rates of lymphocyte proliferation with respect to defined concentration levels of IL-2 and IL-4 and comparing these values to their default amount in the human body before benchmarking with any *in-vitro* or *in-vivo* applications.

While much experimental and computational work has been done to investigate how APCs interact with T cells, the mechanism is still not fully understood in different applications. For example, there is need to understand how pathogen influence APCs connection with different types of T and B cells to effect both innate and adaptive immune responses. Also, the interplay between IL-2 and IL-4 with proinflammatory cytokines (IL-1 β , IL-6, TNF- α ) also requires better understanding. Likewise, a better understanding of how mediating the effect of IL-4 on IL-2 as a strategy to stop pathogens from growing within the body is required. Furthermore, more investigation is needed to determine relevant concentrations of cytokines such as IL-2 and IL-4 during immunotherapy which promotes the activation of immune cells against the invasion of pathogens. Lastly, although not widely covered in this work, studying combination therapy response including immunotherapies within the context of dynamic complex immune-tumor microenvironments is also an interesting future direction. This study uses a stochastic ODE model framework to predict the systematic dynamic immune response resulting from an interplay of lymphocyte activation mediated by APCs and multiple interacting cytokines. Modeling such a complex immune response holds promise for optimizing drug combination therapies and minimizing off-target effects.

## Data Availability

The original contributions presented in the study are included in the article/Supplementary Material, further inquiries can be directed to the corresponding author.
