# Supplementary material for: Mathematical Modeling of Proliferative Immune Response Initiated by Interactions Between Classical Antigen-Presenting Cells Under Joint Antagonistic IL-2 and IL-4 Signaling
Source: Front Mol Biosci. 2022 Jan 28;9:777390. doi: 10.3389/fmolb.2022.777390 (PMC8831889; doi:10.3389/fmolb.2022.777390)
Supplement: Supplementary file 1 [file DataSheet2.pdf]

Supplementary Material

Mathematical modeling of proliferative immune response initiated by interactions between classical antigen presenting cells under joint antagonistic IL-2 and IL-4 signaling

Proliferation of CD4+ T cells in terms of activation of naïve T cell with activated B cells and mature Dendritic cells

Significant clinical research on cell-based vaccines has focused on dendritic cells (DCs), the so-called "professional" APCs because of their efficiency in priming cytotoxic T cells (CTLs), and their highly active extra-cellular protein uptake and antigen-processing capability. Despite these advances, some limitations in the use of DC remain as a platform for clinical use. First, DCs are limited by their relative paucity in human blood (López *et al.*, 2003). Second, harvesting large numbers of DC needed for multiple rounds of vaccinations, particularly for pediatric patients or patients who have undergone extensive myeloablative chemotherapy, may represent a challenge. A limitation in DC growth potential in vitro precludes long term expansion of these populations in culture. Third, it is necessary to generate DC at a specific stage of maturation to promote protective immunity rather than T-cell anergy *(Belz et al.*, 2002;Nouri-Shirazi and Thomson, 2006) or regulatory T-cell activation (Tarbell *et al*., 2007). Furthermore, even the paradigm that fully mature DC always induce immunity has been challenged (Rutella *et al*., 2006). In general, the type of DC and the mode of its activation play critical, sometimes complex roles in determining the ability of these APCs to effectively induce immunity in vivo (Schnurr *et al*., 2005). These challenges have led other cell types to also be considered for cell-based APC vaccines, including B-cells (Pozzi *et al*., 2005;Ahmadi *et al*., 2008).

Due to these considerations, the use of CD40 ligand (CD40L)-stimulated B cells as alternative APC to DC has been suggested (Schultze *et al*., 1997;Coughlin *et al*., 2004). Indeed, recent studies have identified CD40 ligand (CD40L) as the critical membrane-expressed molecule responsible for T cell dependent B-cell activation. CD40L co-operates with various cytokines to induce B-cell activation, proliferation, and immunoglobulin isotype switching (Armitage and Alderson, 1995). CD40L-activated B cells present antigen to either $CD4^+$ or $CD8^+$ T cells following RNA transfection, retroviral transduction, or direct loading of major histocompatibility complex (MHC) class I with peptides (Coughlin *et al*., 2004).

Activated B cells offer three potential advantages over DC: (1) B cells are far more frequent in peripheral blood than DC (10–30% versus 0.5–1% of peripheral white blood cells, respectively) (Van Voorhis *et al*., 1982). (2) Human B-cell populations activated with CD40L and recombinant interleukin-4 (rIL-4) can be expanded for relatively long periods of time in culture, generating hundreds of millions of APCs from small blood volumes in a clinically feasible time frame (Coughlin *et al*., 2004;Schultze *et al*., 2004). (3) B cells express antigen-specific surface receptors which can be exploited to capture and augment presentation of tumor and pathogen-specific antigens. These putative advantages notwithstanding, the efficacy of activated B cells as APC is seldom compared with that of mature DC in head-to-head analyses (Coughlin *et al*., 2004).

Interested in comparing CD40L-activated B cells and mature B cells as APCs, Tahamtan et *al*. (Ahmadi *et al*., 2008) considered different characteristics of activated B cells in the immune system. It includes the ability to expand primary B-cell populations in vitro, enhanced antigen processing by B cells following CD40L signaling, and increased antigen uptake through the BCR, to demonstrate the feasibility of preparing large numbers of efficient, antigen-specific primary B cells for antigen presentation to naïve or primed primary T cells. Accordingly, they implemented experimental design to enable a direct head-to-head comparison between CD40L-activated B cells and mature DC with regard to presentation of MHC class I- and II-restricted peptides and whole protein antigens. Therefore, based on the *in-vivo* experiment performed in (Ahmadi *et al*., 2008) from which we collected the data upon request, the objective of our study is to assess $CD4^+$T cells activation in terms of mature DC and CD40L-activated B cells to enable the comparison of the proliferation of $CD4^+$T cells in terms of both APCs. In the following, we present a resume of the experimental design which fit to the criteria of our study. It includes Animals, Antigens, activation and growth of B cells with CD40L, and the mature DCs. The description of other materials could be found in (Ahmadi *et al*., 2008). Results from the study are also summarized below.

The comparable levels of MHC class I and II molecules and of costimulatory molecules on mature DC and CD40L-activated B cells suggest that these two types of APC may be similar in their ability to present MHC binding peptide antigens that do not require antigen uptake or intracellular processing. To test this hypothesis, CD40L-activated murine B cells, expanded for a minimum of 12 days, and DC were pulsed with MHC class I or MHC class II-binding peptides, and the responses of peptide/MHC-restricted $CD4^+$ and $CD8^+$ T cell clones assayed by 3H-thymidine incorporation. The peptides chosen for these experiments were derived from autoantigens, MBP, as it is anticipated that at least one application of CD40L-activated B cells will be to present self-associated antigens. At APC to responder T-cell ratios of 1:4 and 1:2, CD40L-activated B cells were at least as efficient as DC at presenting the MHC class II-binding self-peptide, MBP (Krasteva *et al*., 1996;Hung *et al*., 2007) to CD4+ T-cell clone 12. These data indicate that primary CD40L expanded B cells are efficient presenters of self-peptides to both $CD4^+$ and $CD8^+$ T cells and ranked CD40L-activated B cells more efficient than mature DCs. Figure S4 shows bar plots comparing CD4+ T cell proliferation levels between CD40L-activated B cells and mature DC with peptide presentation to CD4+ T-cell clones. A higher concentration is observed for B cells.

This is a provisional file, not the final typeset article

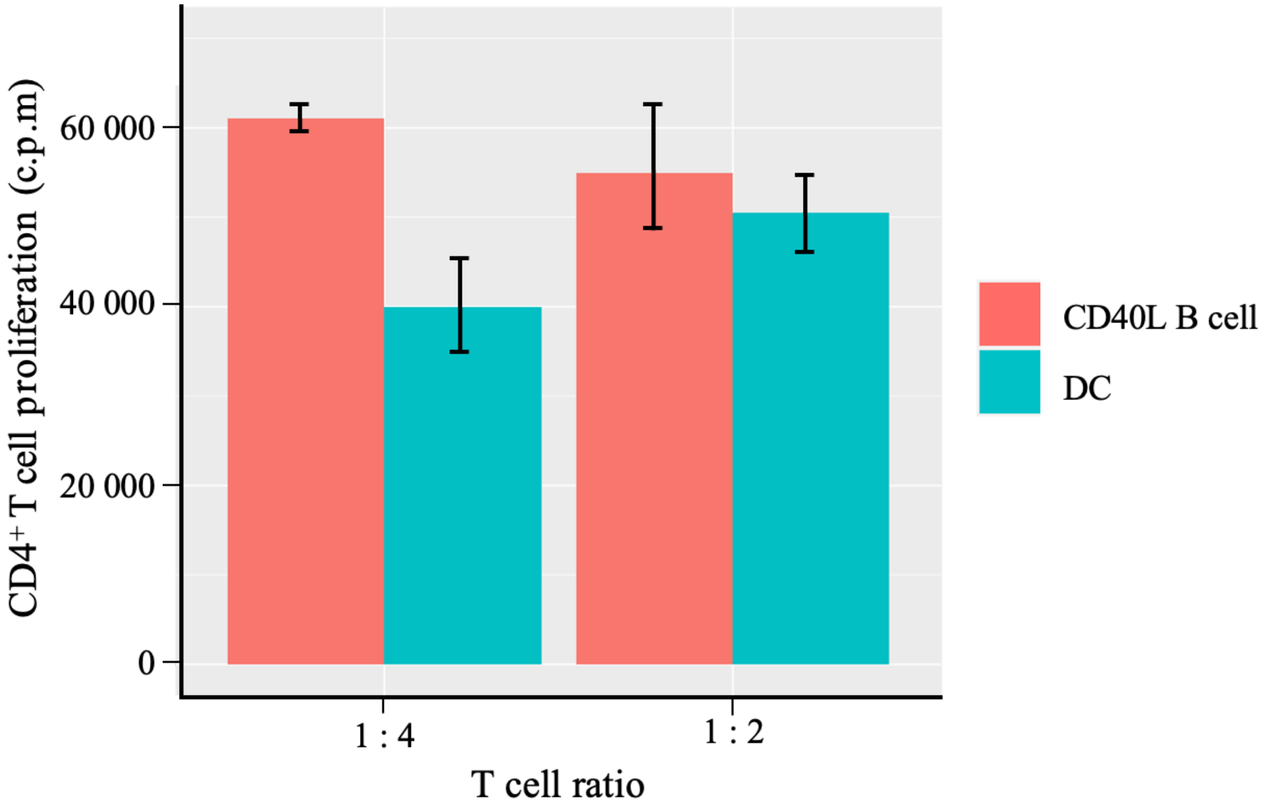

Figure S4. Activated B cells are efficient APCs. Murine CD40L-activated B cells are comparable to mature DC in peptide presentation to CD4+ T-cell clones. Bone marrow DC or splenic CD40L-activated and expanded B-cell populations were generated and loaded with 10 $\mu g/ml$ MBP peptides, irradiated and cultured for 36 hours with MBP/MHC II-restricted CD4+ T cells ('clone 12'). 3H-thymidine was added, and CPM incorporation determined 18–20 hours later. Data obtained from independent cultures of APC are presented as means ± SE. Background incorporation of CD4+ T-cell clone cultured in the presence of APC.

Reference

Ahmadi, T., Flies, A., Efebera, Y., and Sherr, D.H. (2008). CD40 Ligand-activated, antigen-specific B cells are comparable to mature dendritic cells in presenting protein antigens and major histocompatibility complex class I-and class II-binding peptides. *Immunology* 124**,** 129-140.

Armitage, R.J., and Alderson, M.R. (1995). B-cell stimulation. *Current opinion in immunology* 7**,** 243-247.

Belz, G.T., Behrens, G.M., Smith, C.M., Miller, J.F., Jones, C., Lejon, K., Fathman, C.G., Mueller, S.N., Shortman, K., and Carbone, F.R. (2002). The CD8α+ dendritic cell is responsible for inducing peripheral self-tolerance to tissue-associated antigens. *The Journal of experimental medicine* 196**,** 1099-1104.

Coughlin, C.M., Vance, B.A., Grupp, S.A., and Vonderheide, R.H. (2004). RNA-transfected CD40-activated B cells induce functional T-cell responses against viral and tumor antigen targets: implications for pediatric immunotherapy. *Blood* 103**,** 2046-2054.

Gupta, M.K. (1986). Myelin basic protein and demyelinating diseases. *CRC Critical reviews in clinical laboratory sciences* 24**,** 287-314.

Hung, C.-F., Tsai, Y.-C., He, L., and Wu, T. (2007). DNA vaccines encoding Ii-PADRE generates potent PADRE-specific CD4+ T-cell immune responses and enhances vaccine potency. *Molecular Therapy* 15**,** 1211-1219.

Krasteva, M., Peguet-Navarro, J., Moulon, C., Courtellemont, P., Redziniak, G., and Schmitt, D. (1996). In vitro primary sensitization of hapten-specific T cells by cultured human epidermal Langerhans cells—a screening predictive assay for contact sensitizers. *Clinical & Experimental Allergy* 26**,** 563-570.

López, J.A., Bioley, G., Turtle, C.J., Pinzón-Charry, A., Ho, C.S., Vuckovic, S., Crosbie, G., Gilleece, M., Jackson, D.C., and Munster, D. (2003). Single step enrichment of blood dendritic cells by positive immunoselection. *Journal of immunological methods* 274**,** 47-61.

Lutz, M.B., Kukutsch, N., Ogilvie, A.L., Rößner, S., Koch, F., Romani, N., and Schuler, G. (1999). An advanced culture method for generating large quantities of highly pure dendritic cells from mouse bone marrow. *Journal of immunological methods* 223**,** 77-92.

Nouri-Shirazi, M., and Thomson, A.W. (2006). Dendritic cells as promoters of transplant tolerance. *Expert opinion on biological therapy* 6**,** 325-339.

Pozzi, L.-a.M., Maciaszek, J.W., and Rock, K.L. (2005). Both dendritic cells and macrophages can stimulate naive CD8 T cells in vivo to proliferate, develop effector function, and differentiate into memory cells. *The Journal of Immunology* 175**,** 2071-2081.

Raasakka, A., Ruskamo, S., Kowal, J., Barker, R., Baumann, A., Martel, A., Tuusa, J., Myllykoski, M., Bürck, J., and Ulrich, A.S. (2017). Membrane association landscape of myelin basic protein portrays formation of the myelin major dense line. *Scientific reports* 7**,** 1-18.

Rutella, S., Danese, S., and Leone, G. (2006). Tolerogenic dendritic cells: cytokine modulation comes of age. *Blood* 108**,** 1435-1440.

Schnurr, M., Chen, Q., Shin, A., Chen, W., Toy, T., Jenderek, C., Green, S., Miloradovic, L., Drane, D., and Davis, I.D. (2005). Tumor antigen processing and presentation depend critically on dendritic cell type and the mode of antigen delivery. *Blood* 105**,** 2465-2472.

Schultze, J.L., Grabbe, S., and Von Bergwelt-Baildon, M.S. (2004). DCs and CD40-activated B cells: current and future avenues to cellular cancer immunotherapy. *Trends in immunology* 25**,** 659-664.

Schultze, J.L., Michalak, S., Seamon, M.J., Dranoff, G., Jung, K., Daley, J., Delgado, J.C., Gribben, J.G., and Nadler, L.M. (1997). CD40-activated human B cells: an alternative source of highly efficient antigen presenting cells to generate autologous antigen-specific T cells for adoptive immunotherapy. *The Journal of clinical investigation* 100**,** 2757-2765.

Tarbell, K.V., Petit, L., Zuo, X., Toy, P., Luo, X., Mqadmi, A., Yang, H., Suthanthiran, M., Mojsov, S., and Steinman, R.M. (2007). Dendritic cell–expanded, islet-specific CD4+ CD25+ CD62L+ regulatory T cells restore normoglycemia in diabetic NOD mice. *The Journal of experimental medicine* 204**,** 191-201.

Van Voorhis, W.C., Hair, L.S., Steinman, R.M., and Kaplan, G. (1982). Human dendritic cells. Enrichment and characterization from peripheral blood. *The Journal of experimental medicine* 155**,** 1172-1187.

This is a provisional file, not the final typeset article
