# Supplementary material for: Mathematical Modeling of Proliferative Immune Response Initiated by Interactions Between Classical Antigen-Presenting Cells Under Joint Antagonistic IL-2 and IL-4 Signaling
Source: Front Mol Biosci. 2022 Jan 28;9:777390. doi: 10.3389/fmolb.2022.777390 (PMC8831889; doi:10.3389/fmolb.2022.777390)
Supplement: Supplementary file 2 [file DataSheet3.pdf]

Supplementary Material

Mathematical modeling of proliferative immune response initiated by interactions between classical antigen presenting cells under joint antagonistic IL-2 and IL-4 signaling

Effects of cytokines IL-2 and IL-4 on T cells proliferation

Human anti-cytokine antibodies are polyclonal autoantibodies (aAbs) mainly of the IgG class and are either non-neutralizing or neutralizing or both (Schroeder Jr and Cavacini, 2010). Under physiological conditions, anti-cytokine autoantibodies may potentially play a role in the regulation of biological activities of cytokines either by neutralizing excessive cytokine production or by prolonging the half-life of cytokines in circulation by forming cytokine-antibody immune complexes (Fudala *et al.*, 2008). This potential regulatory function is evidenced by the increase in levels of anti-cytokine autoantibodies with increasing amounts of cytokines (Cappellano *et al.*, 2012). Under certain pathological conditions such as in rheumatoid arthritis (RA) or systemic lupus erythematosus (SLE), these aAbs have been observed to rise with decrease in clinical symptoms, suggesting that anti-cytokine autoantibodies may be used as tools to monitor severity or resolution of disease (Graudal *et al.*, 2002;Gupta *et al.*, 2016).

Lymphocyte populations become activated in a specific order during a T cell-dependent immune response (Andersen *et al.*, 2006). In mice injection with foreign antibodies to IgD, result in the activation of B cells by the cross-linking of their membrane (Finkelman *et al.*, 1993). IgD precedes the activation of CD4+T cells (Morris *et al.*, 2000). B cell activation contributes to the subsequent activation of CD4+T cells by enhancing processing of the anti-IgD antibodies and presentation of the processed antibody (Ab) to CD4+T cells specific for Ab-derived determinants (Goroff *et al.*, 1991;Finkelman *et al.*, 1993). It is worth noting that IgD is a monomeric antibody isotype that is expressed in the plasma membranes of immature B-lymphocytes. It is known that IgD signals for B cells to be activated in order for them to participate in the immune defense (Petar *et al.*, 2005).

Relying on the in-vivo experiment realized in (Morris *et al.*, 2000) from which we collected data upon request, the goal of our study is to assess how the concentration of cytokines IL-2 and IL-4 influences not only their productions through activation of $CD4^{+}$T cells, but also to evaluate the proliferation of CD4+T cells in terms of the concentration levels of the cytokine IL-4. We summarize the results of the study in the following. The description of other materials and methods could be found in (Morris *et al.*, 2000).

Due to IL-4 enhanced anti-Ig Ab-induced B cell activation, it has been suspected that the suppressive effect of IL-4 on GαMδ-induced Ab responses might result from inhibition of the CD4+T cell response to GαMδ. The goal of the study is to investigate the effects of IL-4C treatment on T cell IL-2Rα (CD25) and cytokine expression in GαMδ-treated BALB/c mice. Mice sacrificed 4 days after GαMδ injection show a considerable increase in the percentage of CD4+T cells that express CD25. These effects on GαMδ were suppressed by IL-4 (Figure S5A). Also, GαMδ caused considerable increases in IL-2 gene expression by 3 days after injection. However, the treatment with IL-4C inhibited GαMδ-induced IL-

2 responses (Figure S5B) which systematically show a reduction of CD4^{+}T cell proliferation levels since IL-2 is the cytokine growth factor of CD4+T cells (Svetić *et al*., 1991).

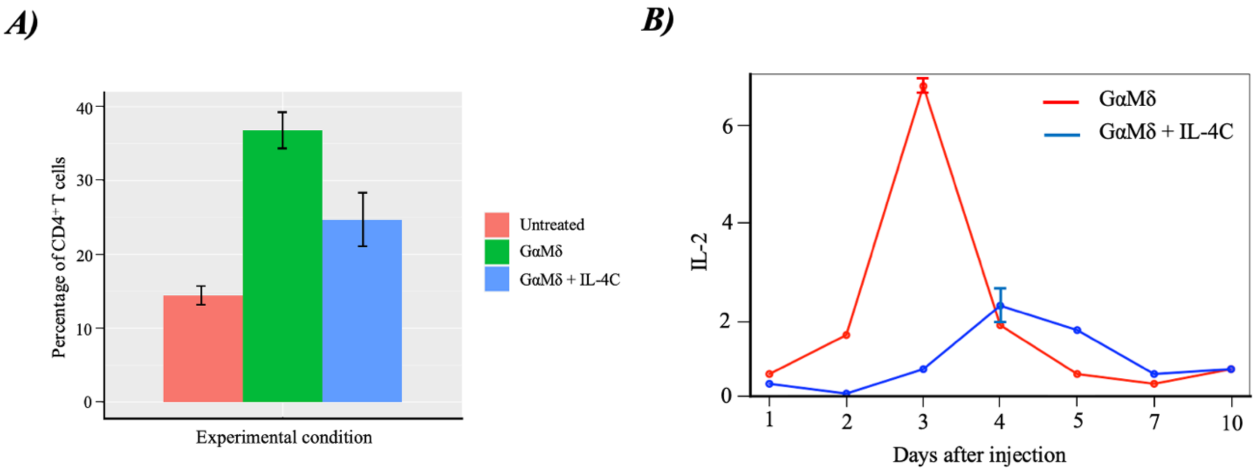

Figure S5: IL-4C inhibits GαMδ induction of CD25 expression by CD4+T cells and IL-4C inhibits gene expressions of IL-2. **A)** (1) BALB/c mice were left untreated (Untreated). (2) BALB/c mice were injected with 800 µg of GαMδ on day 0 (GαMδ treated). (3) BALB/c mice were injected with 800 µg of GαMδ + IL-4C (5 µg of IL-4 + 30 µg of anti-IL-4 mAb) on day 0 (GαMδ + IL-4C treated). Mice were sacrificed on day 4, and spleen cells were stained with FITC-labeled membrane Ab (mAb) GK1.5 (anti-CD4) and biotin-labeled mAb 7D4 (anti-CD25) followed by streptavidin-R-PE. Cells were analyzed by flow microfluorometry. Representative barplots are shown for the fluorescence of CD25 on CD4+ T cells which present arithmetic means and standard error (SE) for percentages of CD4+cells that express CD25. **B)** (1) BALB/c mice were injected with 800 µg of GαMδ on day 0 (GαMδ treated). (2) BALB/c mice were injected with 800 µg of GαMδ + IL-4C (5 µg of IL-4 + 30 µg of anti-IL-4 mAb) on day 0 (GαMδ + IL-4C treated). Mice were sacrificed 1, 2, 3, 4, 5, 7, or 10 days after injection, and splenic cytokine gene expression levels were determined by quantitative RT-PCR. Mice sacrificed on day 1, 2, 7, or 10 days after injection were associated with one experiment, and mice sacrificed on day 3, 4, or 5 days after injection were associated with another. The arithmetic means and standard error (SE) of IL-2 gene expression present a high inhibition effect of IL-4 on the production of IL-2.

Reference

Andersen, M.H., Schrama, D., Thor Straten, P., and Becker, J.C. (2006). Cytotoxic T cells. *Journal of Investigative Dermatology* 126**,** 32-41.

Cappellano, G., Orilieri, E., Woldetsadik, A.D., Boggio, E., Soluri, M.F., Comi, C., Sblattero, D., Chiocchetti, A., and Dianzani, U. (2012). Anti-cytokine autoantibodies in autoimmune diseases. *American journal of clinical and experimental immunology* 1**,** 136.

Finkelman, F.D., Madden, K.B., Morris, S., Holmes, J.M., Boiani, N., Katona, I., and Maliszewski, C. (1993). Anti-cytokine antibodies as carrier proteins. Prolongation of in vivo effects of exogenous cytokines by injection of cytokine-anti-cytokine antibody complexes. *The Journal of Immunology* 151**,** 1235-1244.

Fudala, R., Krupa, A., Stankowska, D., Allen, T.C., and Kurdowska, A.K. (2008). Anti-interleukin-8 autoantibody: interleukin-8 immune complexes in acute lung injury/acute respiratory distress syndrome. *Clinical Science* 114**,** 403-412.

This is a provisional file, not the final typeset article

Goroff, D., Holmes, J., Bazin, H., Nisol, F., and Finkelman, F. (1991). Polyclonal activation of the murine immune system by an antibody to IgD. XI. Contribution of membrane IgD cross-linking to the generation of an in vivo polyclonal antibody response. *The Journal of Immunology* 146**,** 18-25.

Graudal, N., Svenson, M., Tarp, U., Garred, P., Jurik, A., and Bendtzen, K. (2002). Autoantibodies against interleukin 1α in rheumatoid arthritis: association with long term radiographic outcome. *Annals of the rheumatic diseases* 61**,** 598-602.

Gupta, S., Tatouli, I.P., Rosen, L.B., Hasni, S., Alevizos, I., Manna, Z.G., Rivera, J., Jiang, C., Siegel, R.M., and Holland, S.M. (2016). Distinct functions of autoantibodies against interferon in systemic lupus erythematosus: a comprehensive analysis of anticytokine autoantibodies in common rheumatic diseases. *Arthritis & Rheumatology* 68**,** 1677-1687.

Morris, S.C., Gause, W.C., and Finkelman, F.D. (2000). IL-4 suppression of in vivo T cell activation and antibody production. *The Journal of Immunology* 164**,** 1734-1740.

Petar, P., Dubois, D., Rabin, B.S., and Shurin, M.R. (2005). "Immunoglobulin Titers and Immunoglobulin Subtypes," in *Measuring Immunity*. Elsevier), 158-171.

Schroeder Jr, H.W., and Cavacini, L. (2010). Structure and function of immunoglobulins. *Journal of Allergy and Clinical Immunology* 125**,** S41-S52.

Svetić, A., Finkelman, F., Jian, Y., Dieffenbach, C., Scott, D., Mccarthy, K., Steinberg, A., and Gause, W. (1991). Cytokine gene expression after in vivo primary immunization with goat antibody to mouse IgD antibody. *The Journal of Immunology* 147**,** 2391-2397.
