# Supplementary material for: Mathematical Modeling of Proliferative Immune Response Initiated by Interactions Between Classical Antigen-Presenting Cells Under Joint Antagonistic IL-2 and IL-4 Signaling
Source: Front Mol Biosci. 2022 Jan 28;9:777390. doi: 10.3389/fmolb.2022.777390 (PMC8831889; doi:10.3389/fmolb.2022.777390)
Supplement: Supplementary file 3 [file DataSheet1.pdf]

Supplementary Material

Mathematical modeling of proliferative immune response initiated by interactions between classical antigen presenting cells under joint antagonistic IL-2 and IL-4 signaling

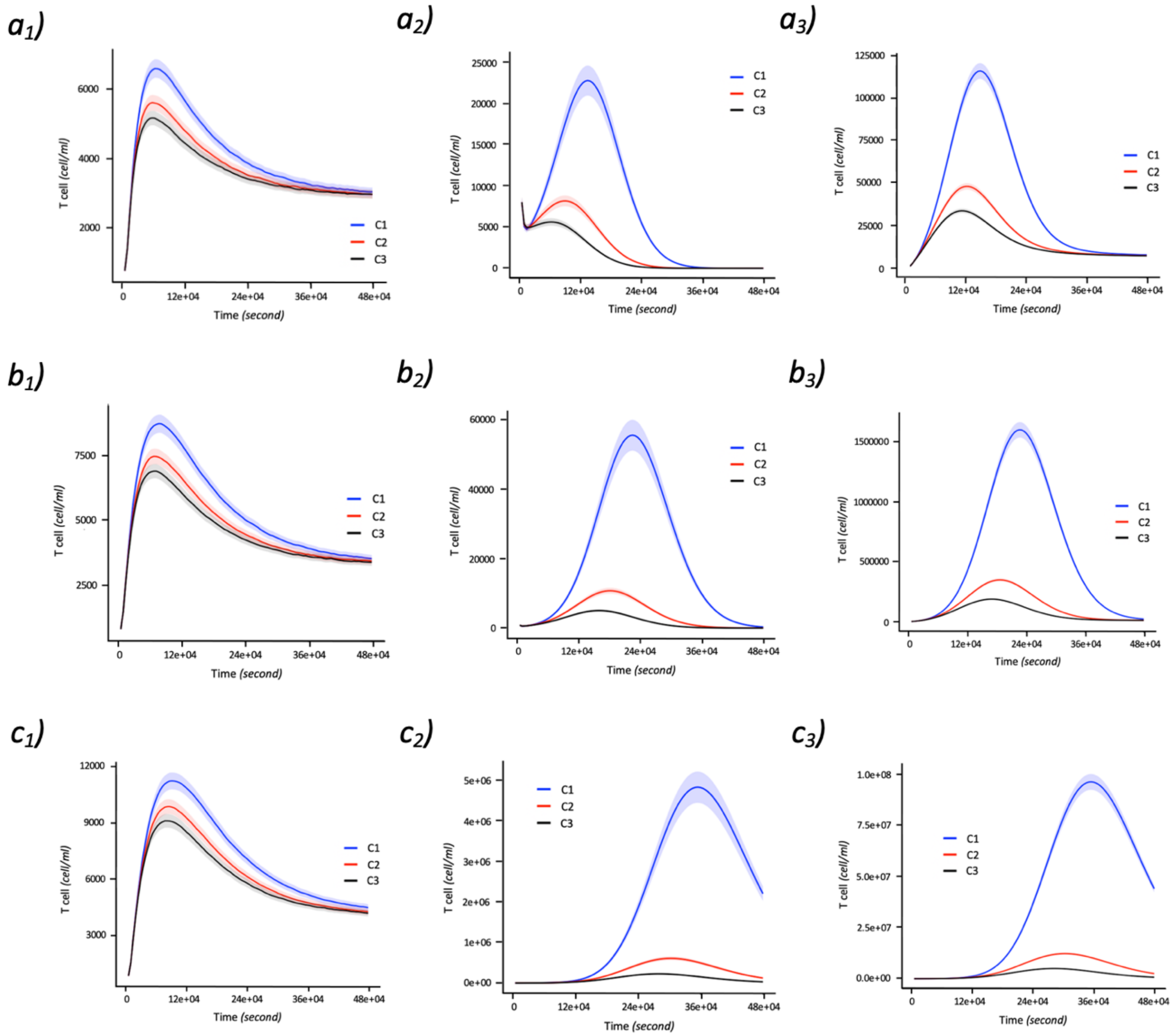

FIGURE S1. Proliferation in terms of changes in number of T cells over time. C1: Initial concentration of IL-4 ($[IL4]_0 = 0.75pM$); **C2**: Second concentration of IL-4 ($[IL4]_1 = 2[IL4]_0$); **C3**: Third concentration of IL-4 ($[IL4]_2 = 3[IL4]_0$). ***a)*** Initial concentration of IL-2 ($[IL2]_0 = 0.75pM$); ***b)*** Second concentration of IL-2 ($[IL2]_1 = 2[IL2]_0$); ***c)*** Third concentration of IL-2 ($[IL2]_2 = 3[IL2]_0$). 1) DC activation by APC 2) B cells activation by APC 3) Joint DC and B cells APC activations. The proliferation of T cell lymphocytes growth with the increase of the concentration of the cytokine IL-2 (c > b > a) and the decrease of the concentration of the cytokine IL-4 (C1 < C2 <C3).

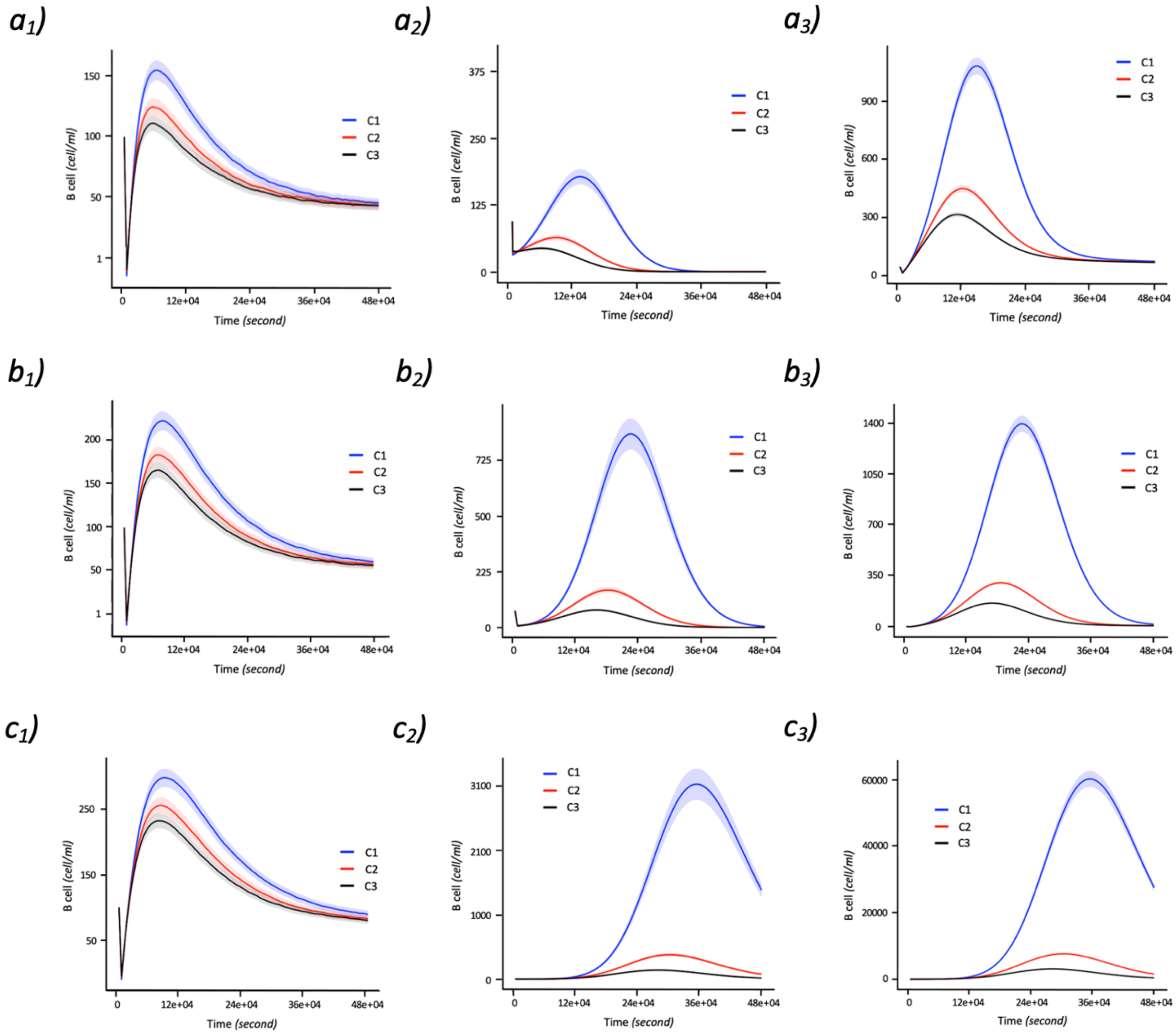

FIGURE S2. Proliferation profile of B cell Lymphocytes over time. C1: Initial concentration of IL-4 ($[IL4]_0 = 0.75pM$); **C2**: Second concentration of IL-4 ($[IL4]_1 = 2[IL4]_0$); **C3**: Third concentration of IL-4 ($[IL4]_2 = 3[IL4]_0$). ***a)*** Initial concentration of IL-2 ($[IL2]_0 = 0.75pM$); ***b)*** Second concentration of IL-2 ($[IL2]_1 = 2[IL2]_0$); ***c)*** Third concentration of IL-2 ($[IL2]_2 = 3[IL2]_0$). 1) DC activation by APC, 2) B cells APC activations, 3) Joint DC and B cells APC activations. The proliferation of B cell lymphocytes growth with the increase of the concentration of the cytokine IL-2 (c > b > a) and the decrease of the concentration of the cytokine IL-4 (C1 < C2 <C3).

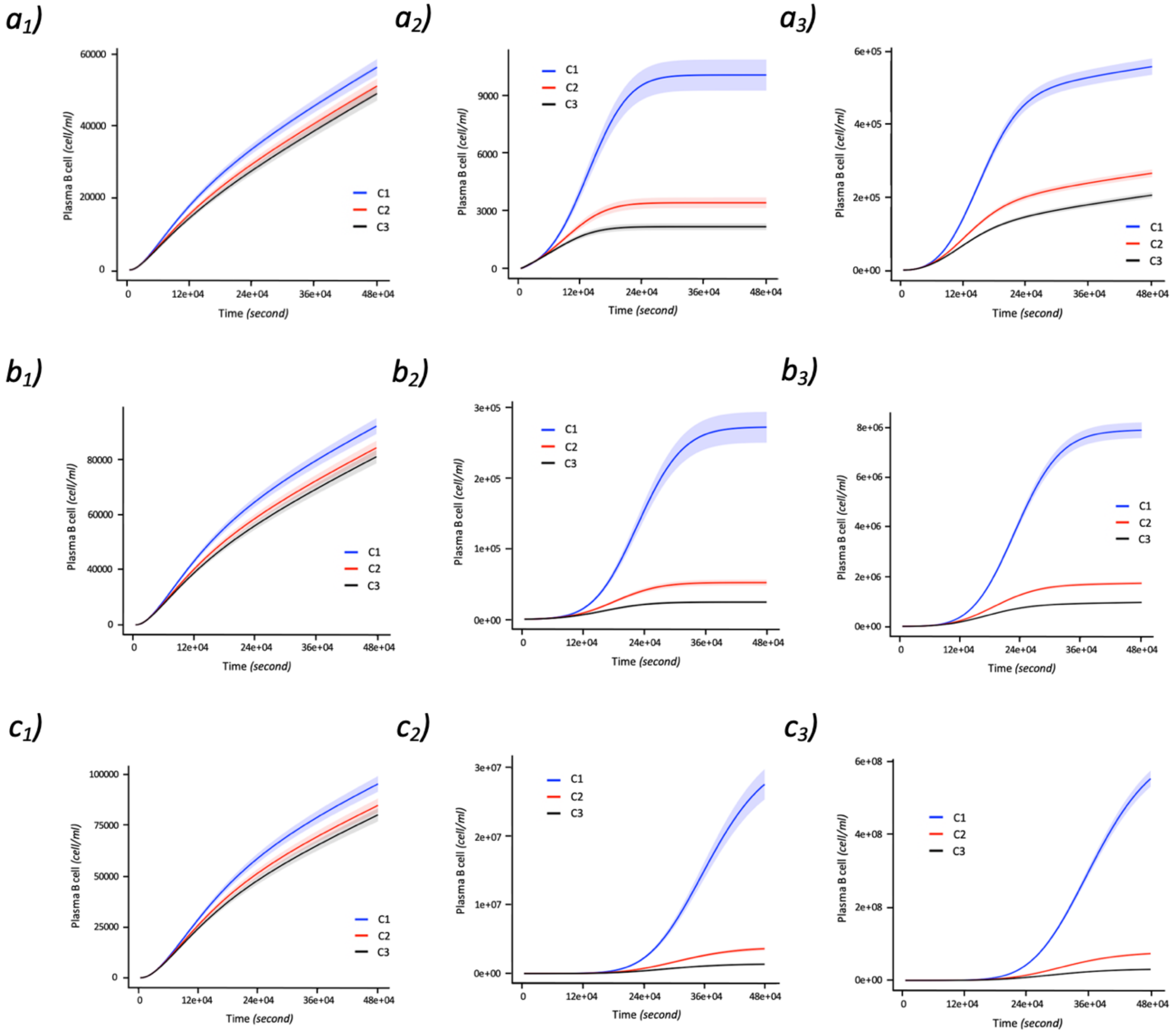

FIGURE S3. Proliferation dynamics of Plasma B cells. C1: Initial concentration of IL-4 ($[IL4]_0$ = 0.75*pM*); **C2**: Second concentration of IL-4 ($[IL4]_1 = 2[IL4]_0$); **C3**: Third concentration of IL-4 ($[IL4]_2 = 3[IL4]_0$). ***a)*** Initial concentration of IL-2 ($[IL2]_0$ = 0.75*pM*); ***b)*** Second concentration of IL-2 ($[IL2]_1 = 2[IL2]_0$); ***c)*** Third concentration of IL-2 ($[IL2]_2 = 3[IL2]_0$). 1) DC APC activation, 2) B cells APC activations, 3) Joint DC and B cells APC activations. The proliferation of Plasma B cell lymphocytes growth with the increase of the concentration of the cytokine IL-2 (c > b > a) and the decrease of the concentration of the cytokine IL-4 (C1 < C2 <C3).
